# NO–IL-6/10–IL-1β axis: a new pathway in steatotic and non-steatotic liver grafts from brain-dead donor rats

**DOI:** 10.3389/fimmu.2023.1178909

**Published:** 2023-08-01

**Authors:** Araní Casillas-Ramírez, Marc Micó-Carnero, Alfredo Sánchez-González, Cristina Maroto-Serrat, Andrés Trostchansky, Carmen Peralta

**Affiliations:** ^1^ Department of Teaching and Research Sub-Direction, Hospital Regional de Alta Especialidad de Ciudad Victoria “Bicentenario 2010”, Ciudad Victoria, Mexico; ^2^ Facultad de Medicina e Ingeniería en Sistemas Computacionales de Matamoros, Universidad Autónoma de Tamaulipas, Matamoros, Mexico; ^3^ Department of Liver, Digestive System and Metabolism, Institut d’Investigacions Biomèdiques August Pi i Sunyer, Barcelona, Spain; ^4^ Departamento de Bioquímica and Centro de Investigaciones Biomédicas (CEINBIO), Facultad de Medicina, Universidad de la República, Montevideo, Uruguay

**Keywords:** brain death, liver transplantation, nitric oxide, steatotic liver grafts, IL-10, IL-6, IL-1, ischemia-reperfusion

## Abstract

**Introduction:**

Brain death (BD) and steatosis are both risk factors for organ dysfunction or failure in liver transplantation (LT)

**Material and methods:**

Here, we examine the role of interleukin 6 (IL- 6) and IL-10 in LT of both non-steatotic and steatotic liver recovered from donors after brain death (DBDs), as well as the molecular signaling pathways underlying the effects of such cytokines.

**Results:**

BD reduced IL-6 levels only in nonsteatotic grafts, and diminished IL-10 levels only in steatotic ones. In both graft types, BD increased IL-1β, which was associated with hepatic inflammation and damage. IL-6 administration reduced IL-1β only in non-steatotic grafts and protected them against damage and inflammation. Concordantly, IL-1β inhibition via treatment with an IL-1 receptor antagonist caused the same benefits in non-steatotic grafts. Treatment with IL-10 decreased IL-1β only in steatotic grafts and reduced injury and inflammation specifically in this graft type. Blockading the IL-1β effects also reduced damage and inflammation in steatotic grafts. Also, blockade of IL-1β action diminished hepatic cAMP in both types of livers, and this was associated with a reduction in liver injury and inflammation, then pointing to IL-1β regulating cAMP generation under LT and BD conditions. Additionally, the involvement of nitric oxide (NO) in the effects of interleukins was evaluated. Pharmacological inhibition of NO in LT from DBDs prompted even more evident reductions of IL-6 or IL-10 in non-steatotic and steatotic grafts, respectively. This exacerbated the already high levels of IL-1β seen in LT from DBDs, causing worse damage and inflammation in both graft types. The administration of NO donors to non-steatotic grafts potentiated the beneficial effects of endogenous NO, since it increased IL-6 levels, and reduced IL-1β, inflammation, and damage. However, treatment with NO donors in steatotic grafts did not modify IL-10 or IL-1β levels, but induced more injurious effects tan the induction of BD alone, characterized by increased nitrotyrosine, lipid peroxidation, inflammation, and hepatic damage.

**Conclusion:**

Our study thus highlights the specificity of new signaling pathways in LT from DBDs: NO–IL-6–IL-1β in non-steatotic livers and NO–IL-10–IL-1β in steatotic ones. This opens up new therapeutic targets that could be useful in clinical LT.

## 1 Introduction

At present, some 80% of hepatic grafts used in liver transplantation (LT) are recovered from donors after brain death (DBDs) (1, 2). It is well known that brain death (BD) of the donor is a factor that drastically increases the chance of a liver graft experiencing preservation/reperfusion injury and decreases graft survival rate (3). In clinical situations, there is a shortage of donors of hepatic grafts for LT (4). In order to reduce the time patients spend waiting for LT, many clinical centers are exploring ways in which to broaden the criteria used to decide if an organ from a so-called marginal donor can be accepted for LT (5, 6). Data from different countries such as USA, France, Australia, or Chile have indicated that as many as 30%–50% of livers recovered from deceased donors are in a state of steatosis, which is one of the key variables that predict negative outcomes after LT (6–10). This is because the risk of hepatic dysfunction and indeed of primary non-function of transplanted liver grafts that are steatotic is greater than that for liver grafts that are not steatotic (11). It is also the case that steatosis is becoming even more prevalent, which means that more steatotic livers are discarded, particularly if the infiltration of fat is severe. Clearly, this exacerbates the current shortage of donors, which has already been described as critical (12). Thus, in order to reduce the LT waiting lists, we need therapeutic strategies that will reduce the post-LT risks of organ dysfunction or failure that are inherent to steatotic livers from DBDs.

The multifunctional cytokine interleukin-6 (IL-6) has been recognized as playing an important role in providing hepatoprotection against warm I/R injury, particularly in reduced-size LT (13, 14). In addition, interleukin-10 (IL-10) has shown protective effects in non-steatotic and steatotic livers that undergo warm ischemia (15, 16). To the best of our knowledge, no study to date has assessed the role of both IL-6 and IL-10 in LT from DBDs. Of interest, a regulation mechanism could exist between IL-6 or IL-10 and interleukin-1 (IL-1), the latter being an interleukin that promotes processes of inflammation and damage (15), including when both steatotic and non-steatotic livers are subjected to warm ischemia-reperfusion (I/R) (15–17). A potential relationship setting up a signaling mechanism between IL-6, IL-10, and IL-1 should not be ruled out, as an interaction between IL-6 and IL-1 has been previously reported in several pathologies (18, 19), and the administration of IL-10 reduced IL-1β levels in both steatotic and non-steatotic livers under warm ischemic conditions (15, 16).

On the other hand, several studies show that nitric oxide (NO) is a crucial mediator of I/R injury in many organs, such as the heart, liver, lung, and kidney (20). NO action decreasing the expression of cytokines has been demonstrated (21–23); for instance, endogenous NO decreased IL-1 hepatic expression in the setting of ischemic preconditioning, thus reducing the deleterious effects of I/R injury (15, 23). In the same way, a relation between IL-10 and IL-6, on the one hand, and NO, on the other, has been reported in several I/R experimental models (15, 24, 25). Thus, in myocardial ischemia, inhibition of NO production resulted in an increase in IL-6 and a reduction in IL-10 (24). In the case of hepatic warm I/R, the therapeutic strategy of ischemic preconditioned triggered NO production, which, in turn, induced IL-10 in non-steatotic and steatotic livers alike (15). In partial LT with steatotic livers, application of venous-systemic oxygen persufflation with NO gas entering the grafts during cold storage caused a reduction in plasma IL-6 levels in the reperfusion period, which was associated with an improvement of graft viability and protection against hepatocellular damage (25).

Taking all this information into account, the current study aimed to evaluate (a) the role of IL-10 and IL-6 in LT of both non-steatotic and steatotic liver recovered from DBDs; (b) the involvement of IL-1 as part of signaling pathways underlying IL-10 and IL-6 effects in this pathology; and (c) if NO might affect the levels of such ILs in LT from DBDs. The results of this study could therefore lead to a description of the molecular signaling mechanisms for both non-steatotic and steatotic livers in LT from DBDs. This, in turn, could result in identifying therapeutic targets that would potentially improve post-LT outcomes in the case of marginal liver grafts recovered from DBDs. Clearly, findings such as these would potentially be of great importance in clinical settings where, as we have said, some 80% of LT grafts are obtained from BD donors, and moreover, it is estimated that hepatic steatosis is present to an important degree in some 30%–50% of deceased donors.

## 2 Materials and methods

### 2.1 Experimental animals

We carried out the study using homozygous (obese: Ob) and heterozygous (lean: Ln) Zucker rats (Iffa Credo, L’Arbresele, France) when they were aged 12 to 14 weeks. In such Zucker rats, steatosis has not been associated with inflammation (26). However, the Ob rats do show severe macrovesicular and microvesicular infiltration of fat into the hepatocytes (>60% steatosis) (27–31), whereas the Ln rats do not show any signs of steatosis (32). We performed all the procedures in accordance with the European Union regulations pertaining to animal experiments (Directive 86/609 EEC).

### 2.2 Experimental design

**1) Sham** (*n* = 12, 6 Ln and 6 Ob). Ob and Ln Zucker rats were anesthetized, ventilated, and maintained normotensive for 6 h (26, 32).
**2) LT** (*n* = 24, 12 transplantations: 6 with non-steatotic grafts and 6 with steatotic grafts). In subgroup 2.1, Ln Zucker rats were anesthetized, ventilated, and maintained normotensive for 6 h. Then, the non-steatotic livers were flushed with University of Wisconsin (UW) solution, isolated, preserved in ice-cold UW solution for 4 h, and implanted into Ln Zucker rats. In subgroup 2.2, the same surgical procedure was repeated, but with Ob Zucker rats as donors and Ln Zucker rats as recipients (26).
**3) BD+LT** (*n* = 24, 12 transplantations: 6 with non-steatotic grafts and 6 with steatotic grafts). In subgroup 3.1, Ln Zucker rats were anesthetized and ventilated. To induce BD, a frontolateral trepanation was performed in the rats and a balloon catheter was introduced in the extradural space (26, 32). The intracranial pressure was increased by inflating the balloon for 1 min, which induced rapid brain injury, leading to immediate BD. Then, the rats were maintained normotensive for 6 h (32–34). Next, the non-steatotic livers were flushed with UW solution, isolated, preserved in ice-cold UW solution for 4 h, and implanted into Ln Zucker rats (26, 32–34). In subgroup 3.2, the same surgical procedure of BD and LT was repeated, but with Ob Zucker rats as donors and Ln Zucker rats as recipients.
**4) BD+IL-1ra+LT** (*n* = 24). Same as group 3, but donors were treated with Anakinra, an IL-1 receptor antagonist (Amgen, Thousand Oaks, CA) at doses of 40 mg/kg (i.v.) just after BD was induced (15, 23, 35).
**5) BD+anti-IL-6+LT** (*n* = 24). Same as group 3, but the donors were treated with an anti-IL-6 antibody (R&D Systems, Minneapolis, MN) at doses of 16.6 µg/kg (i.p.), just after BD was induced (36–38).
**6) BD+anti-IL-10+LT** (*n* = 24). Same as in group 3, but the donors were treated with an anti-IL-10 antibody (Biosource International, Camarillo, CA), at doses of 0.5 mg**/**kg (i.v.) just after BD was induced (15).
**7) BD+anti-IL-6+IL-1ra+LT** (*n* = 12). Same as group 3.1, but the donors were treated with an anti-IL-6 antibody (R&D Systems) at doses of 16.6 µg/kg (i.p.), and with Anakinra, (Amgen) at doses of 40 mg/kg (i.v.) just after BD was induced (15, 23, 35–38).
**8) BD+anti-IL-10+IL-1ra+LT** (*n* = 12). Same as group 3.2, but the donors were with an anti-IL-10 antibody (Biosource International), at doses of 0.5 mg**/**kg (i.v.) and with Anakinra (Amgen) at doses of 40 mg/kg (i.v.) just after BD was induced (15, 23, 35).
**9) BD+IL-6+LT** (*n* = 24). Same as group 3, but the donors were treated with recombinant rat IL-6 (R&D Systems, Minneapolis, MN) at doses of 500 µg/kg (i.p.), 24 and 12 h before BD was induced (14, 39).
**10) BD+IL-10+LT** (*n* = 24). Same as group 3, but the donors were treated with recombinant rat IL-10 (Peprotech EC Ltd., Rocky Hill, NJ) at doses of 10 µg/kg (i.v.), just after BD was induced (15, 40, 41).
**11) BD+IL-6+IL-1+LT** (*n* = 12). Same as group 3.1, but the donors were treated with recombinant rat IL-6 (R&D Systems) at doses of 500 µg/kg (i.p.), 24 and 12 h before BD was induced and with recombinant rat IL-1β (Peprotech EC Ltd.) at doses of 10 µg/kg (i.v.), just after BD was induced (14, 15, 19, 39).
**12) BD+IL-10+IL-1+LT** (*n* = 12). Same as group 3.2, but the donors were treated with recombinant rat IL-10 (Peprotech EC Ltd.) at doses of 10 µg/kg (i.v.) and with recombinant rat IL-1β (Peprotech EC Ltd.) at doses of 10 µg/kg (i.v.), just after BD was induced (15, 19, 39).
**13) BD+NAME+LT** (*n* = 24). Same as group 3, but the donors were treated with N-nitro-L-arginine methyl ester hydrochloride (NAME), an inhibitor of NO synthesis (Sigma Chemical Co., St. Louis, MO) at doses of 10 mg/kg (i.v.) immediately after BD was induced (15).
**14) BD+NO+LT** (*n* = 24). Same as group 3, but the donors were treated with a NO donor, spermine NONOate (Cayman Chemical, Ann Arbor, MI), at doses of 10 mg/kg (i.v.), just after BD was induced (23).

Blood and liver samples were collected at the end of protocols; this was 4 h post-LT. Blood was extracted via the infrahepatic vein in the afternoon and collected in tubes with heparin. Then, blood samples were centrifugated and plasma was immediately frozen in dry ice. Rats were fasted before the extraction. We chose the conditions under which the study was performed, such as doses and the administration times of the pretreatments for the drugs that were used, in accordance with previous studies cited above and also with the results of some preliminary studies we performed. In clinical practice, it is typical for a hepatic donor to be kept in an ICU for a period of 6 h once BD has been diagnosed. During this time, inflammatory alterations tend to occur, which have a negative effect on the quality of the liver, in terms of LT outcomes. A cold ischemia period of 4 h is long enough to induce damage after transplantation in non-steatotic and steatotic liver grafts and to allow high survival at 4 h after reperfusion. For these reasons, we believe that the conditions we used in the experiments that form part of our study were the best based on the available evidence and in order to assess the effects of the different ILs we studied on hepatic damage and also on the molecular signaling pathways of those ILs in both non-steatotic and steatotic livers from DBDs.

Considering that IL-6 and IL-10 might be generated in intestine and adipose tissue in the context of different liver diseases (42–44), we evaluated whether in addition to the liver, such tissues were contributing to the generation of both IL-6 or IL-10 observed in non-steatotic and steatotic liver grafts, respectively, at 4 h post-LT. For this, the levels of IL-1 and IL-6 were measured in adipose tissue and intestine from experimental groups 1, 2, and 3.

To explore at what point in the surgical process cytokines were originated in each type of liver, experiments on the following groups were performed:

**15) CI** (*n* = 12 rats, 6 Ob and 6 Ln Zucker rats): Animals were anesthetized, and livers were subsequently flushed with UW solution, isolated, and maintained in cold ischemia in UW solution for 4 h.
**16) BD** (*n* = 12 rats, 6 Ob and 6 Ln Zucker rats): Animals were anesthetized and ventilated. After BD induction, rats were maintained normotensive with colloid infusion for 6 h.
**17) BD+CI** (*n* = 12 rats, 6 Ob and 6 Ln Zucker rats): Animals were anesthetized and ventilated. After BD induction, rats were maintained normotensive with colloid infusion for 6 h, and then livers were flushed with UW solution, isolated, and maintained in cold ischemia in UW solution for 4 h.

To investigate whether differences in the levels of IL-1β are observed at earlier reperfusion times than 4 h, experiments on the following groups were carried out:

**18) LT 1h** (*n* = 24). Same as group 2, but reperfusion was allowed for 1 h.
**19) BD+LT 1h** (*n* = 24). Same as group 3, but reperfusion was allowed for 1 h.
**20) BD+anti-IL-6+LT 1h** (*n* = 12). Same as group 3.1, but the donors were treated with an anti-IL-6 antibody (R&D Systems, Minneapolis, MN) at doses of 16.6 µg/kg (i.p.), just after BD was induced (27–29), and reperfusion was allowed for 1 h.
**21) BD+anti-IL-10+LT 1h** (*n* = 12). Same as in group 3.2, but the donors were treated with an anti-IL-10 antibody (Biosource International, Camarillo, CA), at doses of 0.5 mg**/**kg (i.v.) just after BD was induced (9), and reperfusion was allowed for 1 h.
**22) LT 2h** (*n* = 24). Same as group 2, but reperfusion was allowed for 2 h.
**23) BD+LT 2h** (*n* = 24). Same as group 3, but reperfusion was allowed for 2 h.
**24) BD+anti-IL-6+LT 2h** (*n* = 12). Same as group 3.1, but the donors were treated with an anti-IL-6 antibody (R&D Systems, Minneapolis, MN) at doses of 16.6 µg/kg (i.p.), just after BD was induced (31–33), and reperfusion was allowed for 2 h.
**25) BD+anti-IL-10+LT 2h** (*n* = 12). Same as in group 3.2, but the donors were treated with an anti-IL-10 antibody (Biosource International, Camarillo, CA), at doses of 0.5 mg**/**kg (i.v.) just after BD was induced (13), and reperfusion was allowed for 2 h.

Liver and blood samples were collected from groups 15 to 25 at the end of the protocol.

### 2.3 Biochemical determinations

We followed standard experimental procedures to determine plasma levels of transaminases [alanine aminotransferase (ALT) and aspartate aminotransferase (AST)]. We established plasma levels of alkaline phosphatase (ALP), the total level of bilirubin, the Von Willebrand factor (vWF), and hyaluronic acid (HA) levels using the appropriate assay kits: colorimetric (ALP: Abcam, Cambridge, United Kingdom) or immunosorbent (Total Bilirubin: MyBioSource Inc., San Diego, CA; vWF: Cusabio, Wuhan, China; HA: R&D Systems, Minneapolis, MN). To determine hepatic edema, we weighed liver samples and then placed them in a drying oven at 55°C until they reached a constant weight. The increase in the wet-to-dry weight ratio represents hepatic edema (45, 46).

Hepatic levels of lipid peroxidation were determined via measurements of malondialdehyde (MDA) formation by using the thiobarbiturate reaction (45, 47). For this, frozen tissue samples were homogenized in 2 ml of Tris buffer at pH 7. For protein precipitation, 0.25 ml of 40% trichloroacetic acid was added to 0.25 ml of homogenate. After mixing and centrifuging at 3,000 *g* for 15 min at 4°C, 0.25 ml of 0.67% thiobarbiturate solution was added to the supernatant, and this mixture was boiled for 15 min. After cooling, optical density was read at 530 nm (48).

To function as an index of the level of accumulation of neutrophils, we determined hepatic levels of myeloperoxidase (MPO) photometrically using a substrate of 3,3′,5,5′-tetramethylbenzidine (45, 47). For this, liver samples were homogenized in phosphate buffer (0.05 M KH_2_PO_4_, pH 6) with 0.5% hexadecyltrimethylammonium bromide (HTBA), then sonicated for 30 s at 20% power. After spending three freeze/thaw cycles in dry ice, the samples were incubated for 2 h at 60°C to eliminate the activity of non-specific peroxidases and MPO inhibitors that could affect the determination. After incubation, the samples were centrifuged for 12 min at 4,000 *g* at 4°C, and the supernatant was recovered. Then, 10 μl of tetramethylbenzidine reagent dissolved in dimethyl sulfoxide at a concentration of 5 mg/ml was added to 5 μl of the supernatant. At time zero (t:0), 70 μl of phosphate buffer (8 mM KH_2_PO_4_, pH 5.4) with 0.05% H_2_O_2_ was added, and the MPO enzymatic kinetics were determined, reading the absorbance for 3 min every 15 s at a wavelength of 630 nm. One enzyme unit was defined as the amount of enzyme that produces an increase of one absorbance unit per minute (47).

We measured hepatic nitrotyrosine to function as a peroxynitrite formation index, using commercial kits (MyBioSource Inc.) (32). For the determination of nitrotyrosine levels in liver tissue, samples were homogenized in 1 ml of 50 mM Na_2_HPO_4_ buffer, pH 7.4, at 4°C, then centrifuged at 20,000 *g* for 30 min at 4°C, and the supernatant was recovered for determination of nitrotirosines (48). cAMP was extracted and quantified with the cAMP enzyme-linked immunosorbent assay (Abcam, Cambridge, United Kingdom) according to the manufacturer’s protocol (49). To obtain liver lysates, frozen tissue was homogenized in 0.5 ml of 0.1 M HCl. After that, lysates were centrifuged at top speed for 5 min and the supernatant was stored at −80°C for subsequent determination of cAMP.

Finally, we measured levels of ILs (IL-1α, IL-1β, IL-6, and IL-10) using enzyme-linked immunosorbent assays, as reported elsewhere, with commercial kits (R&D Systems) (15, 23). To determine IL-1α, IL-1β, and IL-10, frozen liver samples were homogenized in 50 mM phosphate buffer at pH 6, containing 2 mM PMSF, 1 mg/ml antipain, 1 mg/ml leupeptin, and 1 mg/ml pepstatin A. The homogenates were centrifuged at 100,000 *g* for 1 h at 15°C, and the supernatants were stored at −80°C for subsequent determination of interleukins (15). To determine IL-6, liver samples were homogenized in a buffer made up of 50 mM Tris-HCl, 150 mM NaCl, Triton X-100, and a cocktail of protease inhibitors (Complete mini, Merck, Darmstadt, Germany). The homogenate was centrifuged at 3,000 *g* for 15 min at 4°C. Finally, the supernatant was recovered to perform the IL-6 measurement (50).

### 2.4 Histology

Before performing experiments in experimental groups, we determined steatosis level in biopsy specimens of liver tissue from lean and obese Zucker rats (*n* = 12, 6 Ln and 6 Ob). Since an association between liver fibrosis and adverse LT outcome has been reported (L4), assessment of fibrosis was also performed in Zucker rats. The removed livers were immediately fixed in a 10% phosphate buffered formalin solution and then embedded in paraffin. Samples were cut and stained with hematoxylin–eosin (HE) stain reagent for light microscopic observation. Histopathological findings were evaluated by scoring methods according to the report of Kleiner et al. (51) through a microscope (Nikon, Tokyo, Japan) at ×100 and ×200 magnification. Steatosis was graded 0–3 according to the percentage of cells with fatty droplets (0, <5%; 1, 5%–33%; 2, >33%–66%; 3, >66%). Lobular inflammation was graded 0–3 according to overall assessment of all inflammatory foci (0, no foci; 1, <2 foci per ×200 field; 2, 2–4 foci per ×200 field; 3, >4 foci per ×200 field). Liver cell injury was graded 0–2 according to ballooning degeneration (0, none; 1, few balloon cells; 2, many cells/prominent ballooning). Fibrosis was graded 0–4 according to the extent of fibrosis (0, no fibrosis; 1, zone 3 fibrosis; 2, zone 3 and portal fibrosis; 3, zone 3 and portal fibrosis with bridging fibrosis; and 4, cirrhosis). These scores were added, and the sum was used as a histopathological score (52–55). This assessment was carried out by three independent researchers who were unaware of the experimental conditions. Average values of the three mean scores assessed by these researchers were then used as a histopathological score for each animal. The pathological findings of obese Zucker rats used in the study were classified into severe simple steatosis with neither inflammation nor fibrosis, and histological findings of lean Zucker rats were classified as normal. This difference of steatosis in the Ob versus Ln Zucker rats was confirmed by using specific lipid staining such as red oil staining. Liver tissue was frozen and red oil O staining was used according to standard procedures. The hepatic steatosis was determined by quantifying cells with stained lipid droplets in 30 randomly chosen high-power fields per section. Data were expressed as the percentage of stained cells with respect to the total number of hepatocytes. Ob Zucker rats showed severe and macrovesicular and microvesicular fatty infiltration in hepatocytes. In contrast, Ln Zucker rats show no evidence of steatosis (15, 45, 56). In addition, we also evaluated fibrosis presence or absence with Sirius Red staining in paraffin-embedded liver samples, as this staining is one of the best understood techniques able to selectively highlight collagen networks (57). The percent area of collagen deposition was calculated as collagen area/total area − vascular lumen area × 100 (26). Ln and Ob Zucker rats showed no evidence of fibrosis, which is in agreement with reports describing that Ob Zucker rats show resistance to developing fibrosis (58, 59).

In liver samples obtained from all experimental group studies, we assessed the degree of liver injury also by staining paraffin-embedded liver sections with H&E and then using a method of counting points to determine a histological score (47). An experienced pathologist blinded to the allocation of animals into different experimental groups reviewed biopsy specimens and scored the histologic findings according to the following scale: grade 0, zero or minimal evidence of injury; grade 1, the injury is mild, showing cytoplasmic vacuolation and focal nuclear pyknosis; grade 2, the injury is moderate ranging to severe with extensive nuclear pyknosis, cytoplasmic hypereosinophilia, and the loss of intercellular borders; grade 3, severe necrosis showing disintegration of the hepatic cords, hemorrhage, and neutrophil infiltration; and finally grade 4, very severe necrosis exhibiting disintegration of the hepatic cords, hemorrhaging, and neutrophil infiltration (41, 45).

### 2.5 Statistics

In all determinations represented in figures, biological replicates were used. That is, in each experimental group that was represented in the graphs, samples from six different transplants with the same treatment were included. Data are expressed as mean ± standard error of mean. We applied non-parametric Kruskal–Wallis tests to determine statistical significance when treating differing variables. In contrast, we used Mann–Whitney *U* tests for groups that showed significant differences, and we calculated adjusted *p*-values by the false discovery rate (FDR) method (*p* < 0.05 was considered significant).

## 3 Results

### 3.1 The role played by IL-6 and IL-10 in LT of non-steatotic and steatotic grafts recovered from DBDs

First, we determined the levels of endogenous IL-6 and IL-10 in steatotic and non-steatotic LT from DBDs.

**Figure 1:**
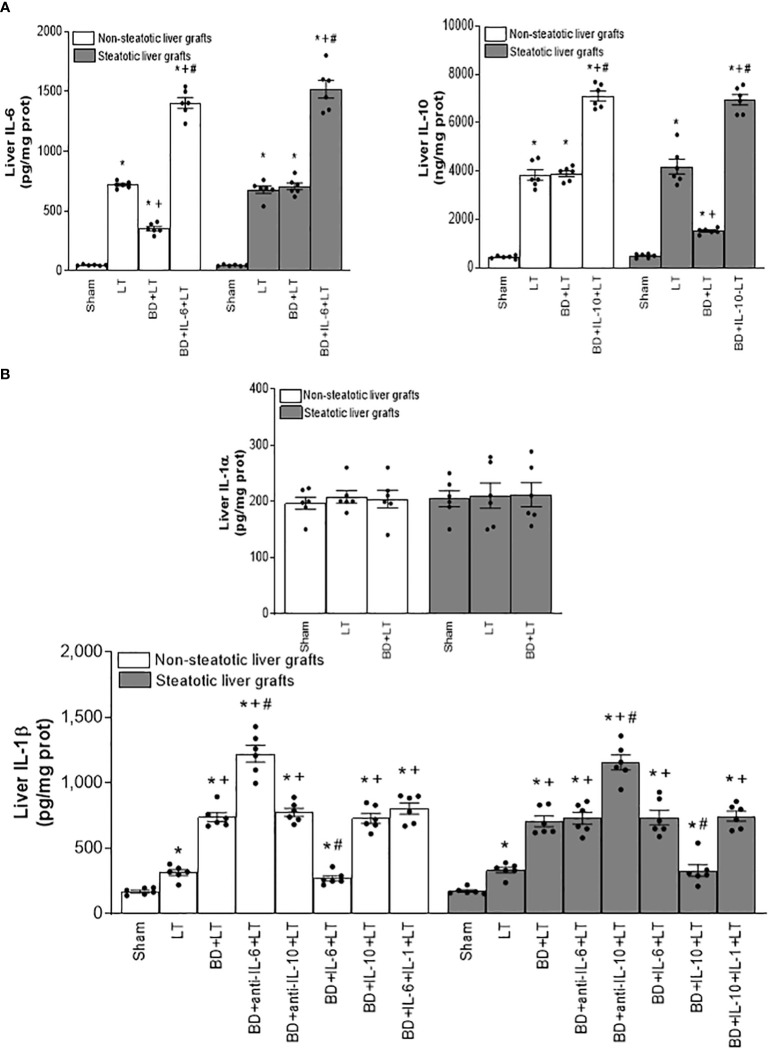
Interleukin levels in steatotic and non-steatotic LT from DBDs. **(A)** Levels of IL-6 and IL-10, and **(B)** levels of IL-1α and IL-1β, all in liver tissue. For A and B, six transplants per group in each measurement. *p < 0.05 versus Sham; ^+^ *p* < 0.05 versus LT; ^#^ *p* < 0.05 versus BD+LT.

Concerning alterations in anti-inflammatory interleukins (ILs), we observed increased levels of IL-6 and IL-10 in LT of both non-steatotic and steatotic livers, compared to the results of the Sham group (**Figure 1A**). The induction of BD (BD+LT) resulted in IL-6 levels in non-steatotic grafts lower than those observed in the LT group. However, such an effect was not observed in steatotic grafts, since in this type of liver, the IL-6 levels were similar in BD+LT and LT groups. With regard to IL-10, inducing BD (BD+LT) resulted in no changes for this interleukin in non-steatotic livers when compared to the LT group. However, when steatosis was present, we observed reduced levels of IL-10 in the BD+LT group, compared to the LT group (**Figure 1A**). Thus, depending on the presence or absence of steatosis, induction of BD downregulated different anti-inflammatory interleukins: IL-6 for non-steatotic and IL-10 for steatotic grafts.

**Figure 2:**
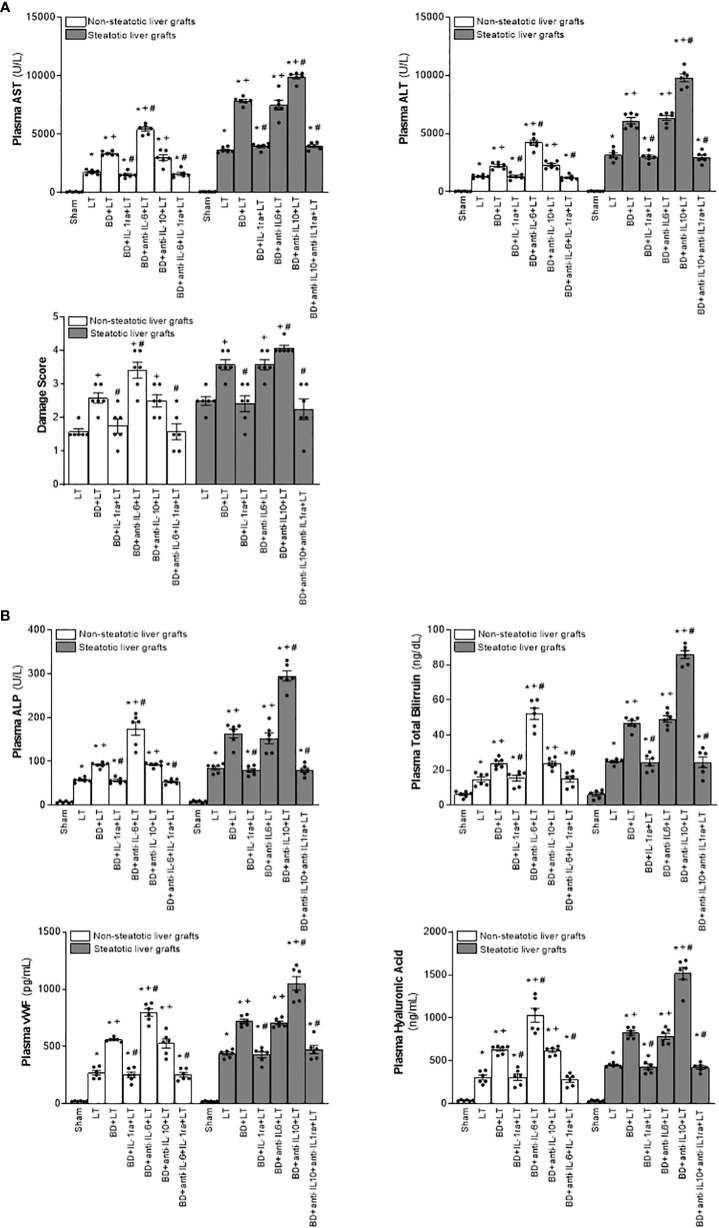
Relevance of endogenous interleukins for damage in steatotic and non-steatotic LT from DBDs. **(A)** ALT and AST levels in plasma and liver damage score. **(B)** ALP, total bilirubin, vWF, and HA levels in plasma. For A and B, six transplants per group in each measurement. *p < 0.05 versus Sham; +*p* < 0.05 versus LT; #*p* < 0.05 versus BD+LT.

**Figure 3:**
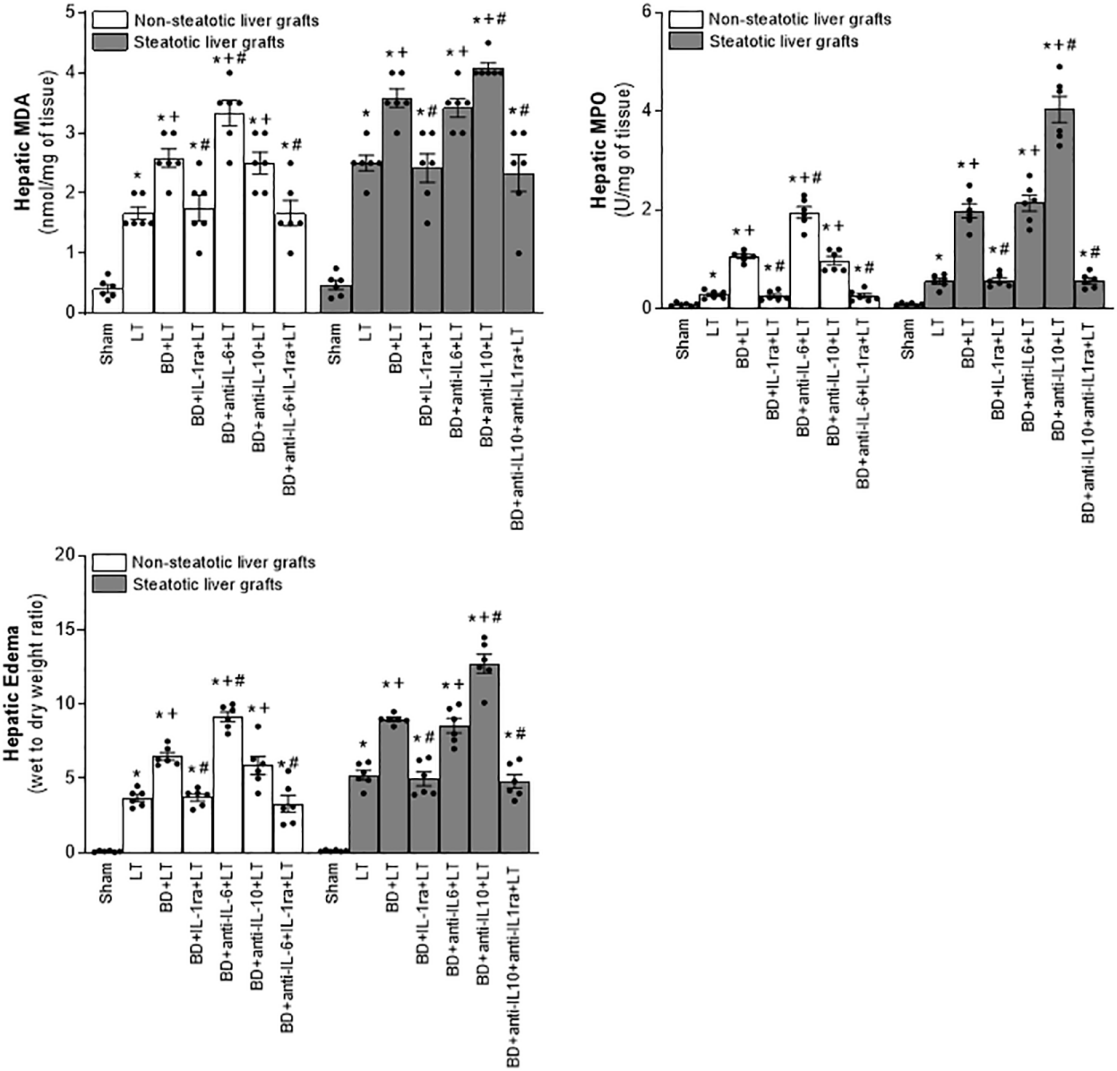
Effect of endogenous interleukins on inflammation in steatotic and non-steatotic LT from DBDs. Hepatic levels of MDA, MPO, and hepatic edema. For A and B, six transplants per group in each measurement. *p < 0.05 versus Sham; ^+^ *p* < 0.05 versus LT; ^#^ *p* < 0.05 versus BD+LT.

Then, we assessed the role played by endogenous IL-6 and IL-10. To this end, we inhibited the action of either IL-6 or IL-10, always in liver grafts from DBDs, and evaluated the effects on inflammation and damage. The administration of antibodies against IL-6 (BD+anti-IL-6+LT) raised parameters of damage (levels of transaminases, damage scores, ALP, total bilirubin, and also endothelial cell damage determined by vWF and HA levels) and inflammation (degree of neutrophil accumulation as measured by MPO, edema formation, and oxidative stress assessed using MDA) in non-steatotic grafts from DBDs, with respect to the results of the BD+LT group (**Figures 2**, **3**). On the other hand, in steatotic liver grafts, no changes were observed in parameters of inflammation and damage (**Figures 2**, **3**) when comparing the results of the BD+anti-IL-6+LT group to those of the BD+LT group. When we addressed the effects of endogenous IL-10, we observed that this cytokine was protective only in steatotic grafts since the administration of antibodies against IL-10 exacerbated hepatic damage and inflammation in steatotic liver grafts from DBDs, in comparison with LT from DBDs without treatment (that is, BD+anti-IL-10+LT vs. BD+LT) (**Figures 2**, **3**). On the other hand, in the absence of steatosis, we observed that levels of these same parameters reflecting damage and inflammation were similar in the BD+anti-IL-10+LT group and in the BD+LT group. Thus, these results indicate that the protection offered by endogenous IL-6 and IL-10 is specific, according to the liver type: IL-6 provides protection in non-steatotic grafts and IL-10 in steatotic ones, within the context of LT from DBDs.

**Figure 4:**
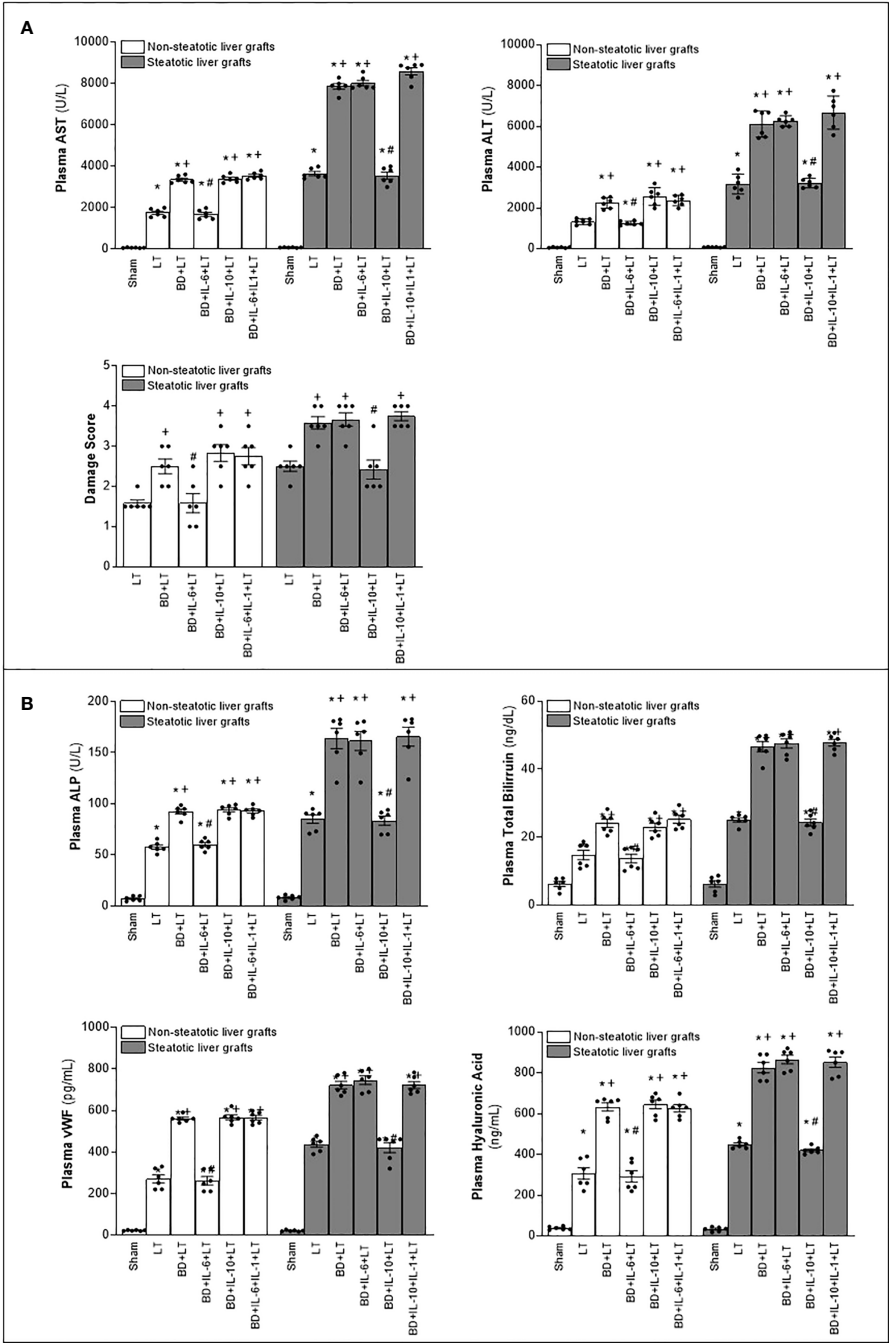
Relevance of exogenous IL-6 and IL-10 for damage in steatotic and non-steatotic LT from DBDs. **(A)** ALT and AST levels in plasma and liver damage score. **(B)** ALP, total bilirubin levels, vWF, and HA levels in plasma. For A and B, six transplants per group in each measurement. *p < 0.05 versus Sham; ^+^ *p* < 0.05 versus LT; ^#^ *p* < 0.05 versus BD+LT.

**Figure 5:**
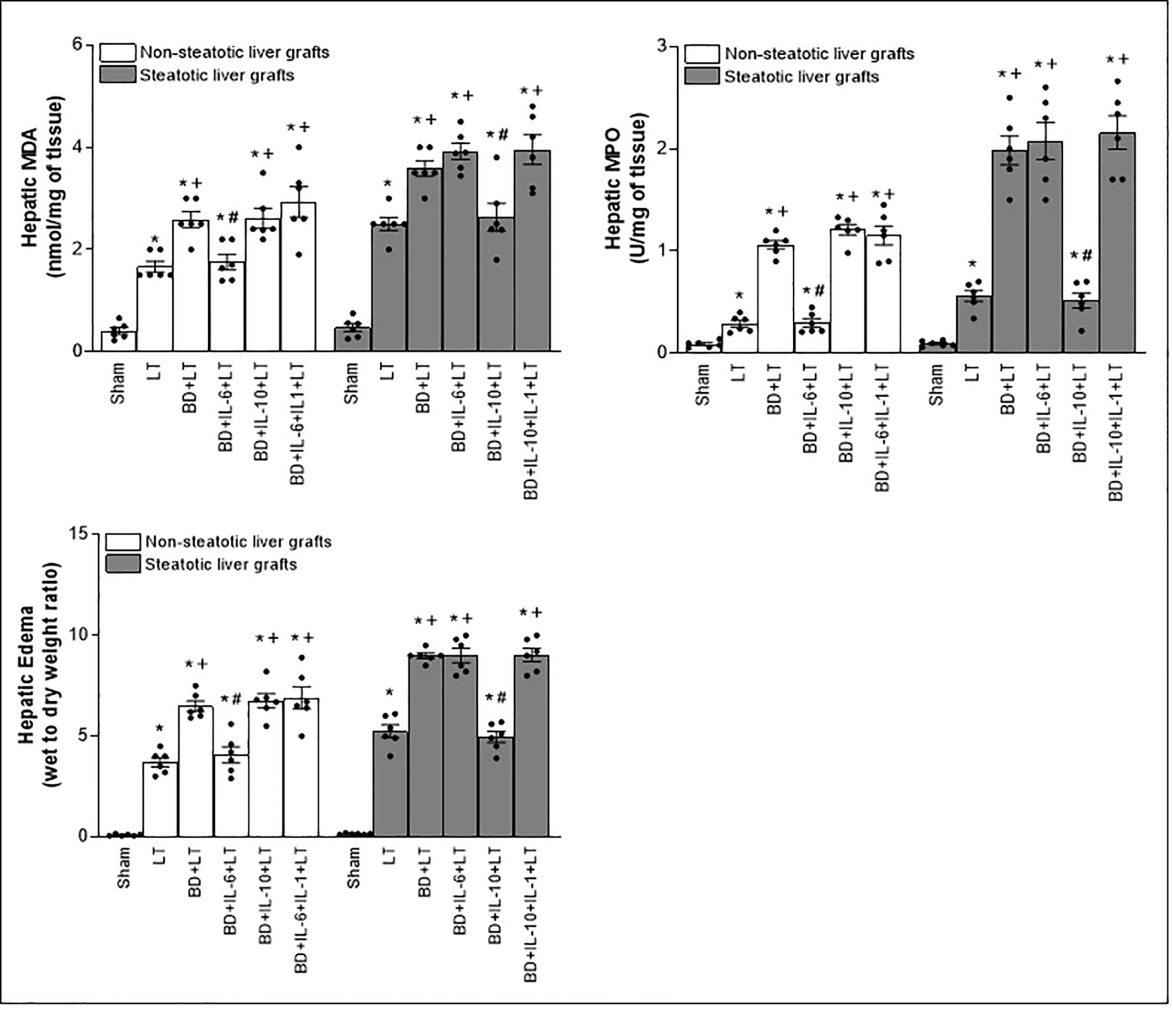
Effect of exogenous IL-6 and IL-10 on inflammation in steatotic and non-steatotic LT from DBDs. Hepatic levels of MDA, MPO, and hepatic edema (six transplants per group in each measurement). *p < 0.05 versus Sham; ^+^ *p* < 0.05 versus LT; ^#^ *p* < 0.05 versus BD+LT.

In consideration of the protection provided by endogenous IL-6 and IL-10 in LT from DBDs, we decided to evaluate whether treatments based on the administration of exogenous IL-6 and IL-10 might potentiate the benefits induced by the former regarding hepatic damage and inflammation in non-steatotic and steatotic livers, respectively. The administration of exogenous IL-6 (BD+IL-6+LT) increased IL-6 levels in both types of livers (**Figure 1A**) but livers obtained from DBDs were only protected against damage and inflammation in the absence of steatosis. In fact, for the BD+IL-6+LT group with non-steatotic livers, the liver injury parameters (levels of transaminases, damage scores, ALP, levels of total bilirubin, vWF, and HA; **Figure 4**) and inflammation indicators (MPO, edema, and MDA; **Figure 5**) were lower than those of the BD+LT group, while no changes were observed in those same experimental groups (BD+IL-6+LT and BD+LT) when steatotic grafts were used, when they were compared. The exogenous IL-10 supplementation (BD+IL-10+LT) increased IL-10 in non-steatotic and steatotic liver grafts (**Figure 4**); however, it did not abrogate the rise in parameters, indicating hepatic damage and inflammation in grafts obtained from DBDs in the absence of steatosis compared with the results of the BD+LT group (**Figures 4**, **5**). In contrast, this intervention was effective as a therapeutic strategy in the presence of steatosis, since, in the BD+IL-10+LT treatment group, we observed reduced transaminase levels, damage scores, ALP, total levels of bilirubin, vWF, HA, MPO, edema, and MDA in grafts in the presence of steatosis with respect to the results of the BD+LT group (**Figures 4**, **5**). Therefore, the benefits from exogenous IL-6 and IL-10 seem to be dependent on the type of liver: IL-6 was useful in non-steatotic livers and IL-10 was useful in steatotic ones. The dose of IL-6 and IL-10 used in the current study was the most effective because our preliminary results indicated that if these doses were increased threefold, this was not associated with more protection against damage (data not shown).

### 3.2 Protective mechanisms of IL-6 and IL-10 in LT of non-steatotic and steatotic grafts recovered from DBDs

Considering previous publications that reported a regulatory role of IL-6 and IL-10 on IL-1 (11, 12, 14, 15), IL-1 was investigated as a possible mediator involved in signaling mechanism underpinning IL-6 and IL-10 beneficial effects. For this, hepatic levels of IL-1α and IL-1β in LT and BD surgical settings were first determined. Our results indicated that IL-1α does not mediate I/R injury in liver. This is because we observed levels of IL-1α in both non-steatotic and steatotic grafts obtained from DBDs (BD+LT), as well as in those grafts used in LT that had been obtained from donors that had not undergone BD (LT), similar to those of IL-1α in the Sham group (**Figure 1B**). Notwithstanding this, we did observe significant increases in levels of hepatic IL-1β in both grafts that were steatotic and those that were non-steatotic in the BD+LT group, compared to either the Sham group or the LT group (again, see **Figure 1B**). Then, the involvement of IL-1β in the damaging effects induced by BD in both types of liver grafts was evaluated. When we administered an IL-1 receptor antagonist (BD+IL-1ra+LT), we observed that damage in both types of liver was attenuated since, when compared to the BD+LT group, and all the following parameters were reduced: levels of transaminases, damage scores, ALP, total bilirubin, and endothelial cell damage (as determined by vWF and HA levels) (**Figure 2**). In conjunction with these effects, we also observed a reduced inflammatory response in both steatotic and non-steatotic grafts in the BD+IL-1ra+LT group compared to those of the BD+LT group, which was indicated by lower degrees of neutrophil accumulation (as measured by MPO), edema formation, and oxidative stress (as measured using MDA) (**Figure 3**). Thus, we observed that both in the presence and in the absence of steatosis, inhibition of IL-1 action was an effective strategy to reduce injury.

After demonstrating that IL-1β was involved in liver damage in LT with DBDs, we next evaluated whether IL-1β would be acting as a downstream mediator of IL-6 or IL-10. For this, we inhibited the action of either IL-6 or IL-10 in liver grafts from DBDs, to evaluate the effect of these endogenous cytokines on IL-1β. The administration of antibodies against IL-6 (BD+anti-IL-6+LT) increased IL-1β in non-steatotic grafts from DBDs, compared to those from the BD+LT group (this is shown in **Figure 1B**). In contrast to these findings, in grafts from steatotic livers, we found no changes in the levels of IL-1β when we compared the BD+anti-IL-6+LT group to the BD+LT group. Regarding endogenous IL-10, the administration of antibodies against IL-10 increased IL-1β in steatotic liver grafts from DBDs, in comparison with LT from DBDs without treatment (that is, BD+anti-IL-10+LT vs. BD+LT), whereas the levels of IL-1β in liver grafts where there was an absence of steatosis were found to be similar in the BD+anti-IL-10+LT group and in the BD+LT group (**Figure 1B**). In addition, we investigated whether the benefits of exogenous IL-6 in non-steatotic livers and IL-10 in steatotic ones might be explained by their effects on IL-1β. In this sense, administration of IL-6 (BD+IL-6+LT) resulted in reduced IL-1β levels only in non-steatotic livers, whereas treatment with IL-10 (BD+IL-10+LT) led to reduced IL-1β levels only in steatotic livers (**Figure 1B**).

The above results showed that there is a relationship between IL-6 and IL-1β in non-steatotic grafts and between IL-10 and IL-1β in steatotic grafts, always from DBDs. Then, the relevance of such signaling pathways in liver inflammation and damage was investigated. For this, we evaluated whether the increased IL-1β levels were responsible for the exacerbated damage induced by the inhibition of either IL-6 or IL-10 action in liver grafts from DBDs, in the absence and presence of steatosis, respectively. We also found that, whereas blockade of IL-6 (BD+anti-IL-6+LT) increased IL-1β and damage in non-steatotic livers from DBDs more than in the BD+LT group, the inhibition of the action of both IL-6 and IL-1β (BD+anti-IL-6+IL-1ra+LT) gave rise to parameters that reflected liver damage and inflammation (**Figures 2**, **3**), which were lower than those of the BD+LT group. In steatotic grafts from DBDs, suppression of IL-10 (BD+anti-IL-10+LT) increased IL-1β and the parameters of hepatic damage and inflammation when compared to the results in the BD+LT group, but no such effect was observed when both IL-10 and IL-1β effects were suppressed (BD+anti-IL-10+IL-1ra+LT). Indeed, the parameters of hepatic damage and inflammation in steatotic grafts from DBDs were decreased in the BD+anti-IL-10+IL-1ra+LT group, when we compared them with those of the experimental group BD+LT (**Figures 2**, **3**). All of this confirms that in LT and BD, IL-6 in non-steatotic or IL-10 in steatotic livers reduces IL-1β levels, which is necessary to limit liver injury in each type of liver. If IL-6 in non-steatotic livers or IL-10 in steatotic ones is inhibited, IL-1β is increased and liver damage is more exacerbated than is seen with BD+LT. If IL-6 in non-steatotic grafts or IL-10 in steatotic grafts is inhibited but the action of IL-1β is also inhibited, there is no aggravated liver damage.

Next, we investigated whether the benefits of exogenous IL-6 in non-steatotic livers and IL-10 in steatotic ones might be explained by their effects on IL-1β. As previously described, administration of IL-6 (BD+IL-6+LT) resulted in reduced IL-1β levels and liver injury only in non-steatotic livers, whereas treatment with IL-10 (BD+IL-10+LT) led to reduced IL-1β levels and hepatic damage only in steatotic livers. In non-steatotic grafts from DBDs, IL-1β administration to the BD+IL-6+LT group (BD+IL-6+IL-1β+LT) increased IL-1β levels (**Figure 1B**) and this totally reversed the benefits seen from administration of IL-6 on hepatic damage and inflammation (**Figures 4**, **5**). Similarly, in steatotic grafts from DBDs, IL-1β supplementation in the BD+IL-10+LT group (BD+IL-10+IL-1β+LT) raised IL-1β levels and abolished the benefits induced by IL-10, concerning liver injury and inflammatory response (**Figures 1B**, **4**, **5**). This demonstrates that IL-1β is a downstream mediator of IL-6 and IL-10 and its regulation is crucial as part of the signaling mechanisms to achieve the beneficial effects of IL-6 in non-steatotic grafts and IL-10 in steatotic ones when both are subjected to BD+LT.

**Figure 6:**
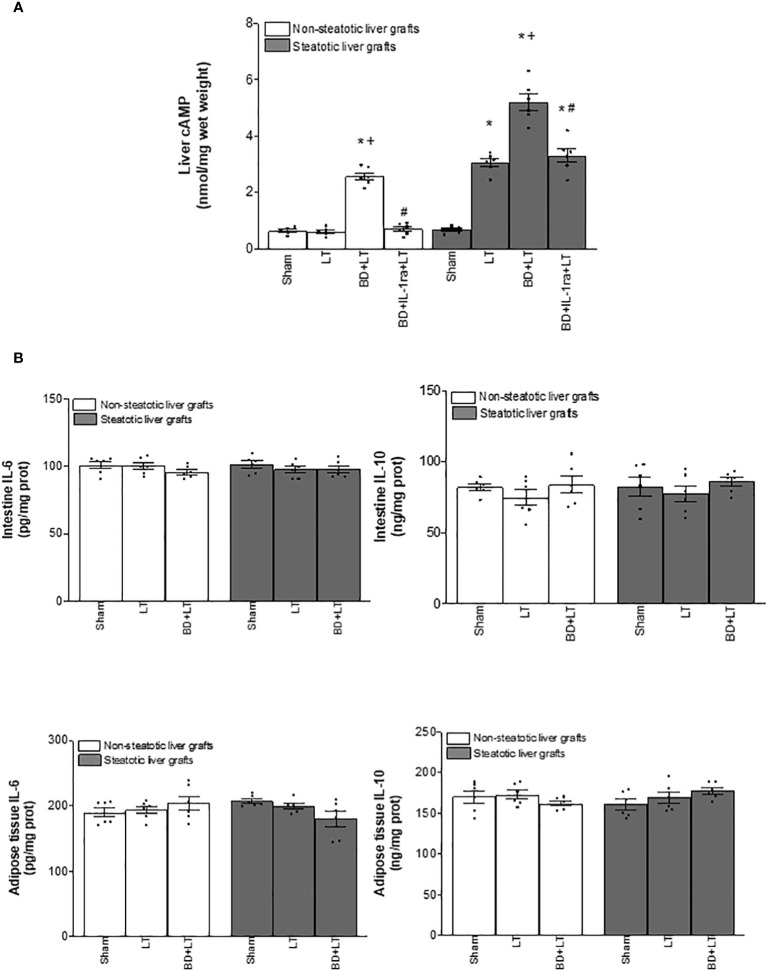
Effects of IL-1β on cAMP **(A)** and source of IL-6, IL-10, and IL-β **(B)** in steatotic and non-steatotic LT from DBDs. **(A)** Hepatic levels of cAMP. **(B)** IL-6, IL-10, and IL-1β in intestine and adipose tissue. For A and B, six transplants per group in each measurement. *p < 0.05 versus Sham; ^+^ *p* < 0.05 versus LT; ^#^ *p* < 0.05 versus BD+LT.

To delve into the IL-6/IL-1β and IL-10/IL-1β signaling mechanism, we also investigated the IL-1β downstream mediator. Cyclic adenosine 3′,5′-monophosphate (cAMP) is produced by adenylate cyclase and acts as an intracellular second messenger for IL-1β (60, 61). In fact, it has been reported that IL-1β increased cAMP (61). When we measured hepatic cAMP levels, our results indicated that cAMP was higher in steatotic and non-steatotic livers of the BD+LT group when compared with the LT group. Of interest, the levels of cAMP were higher in experimental groups with steatotic livers than in those with non-steatotic ones. The inhibition of IL-1β effects through administration of IL-1β receptor antagonist (BD+IL-1ra+LT) reduced cAMP levels in both types of livers (**Figure 6A**). Then, blockade of IL-1β action and diminished hepatic cAMP were associated with a reduction in all parameters of hepatic damage and inflammation evaluated in the current study. Noticeably, the hepatic levels of cAMP induced by IL-1β were in parallel with the levels of liver damage observed in each type of graft; that is, steatotic livers subjected to LT or BD+LT have higher cAMP levels, compared to the levels recorded in non-steatotic livers. Hence, IL-1β regulates cAMP generation, and suppression of these both mediators downstream IL-6 or IL-10 was responsible for the protective effects of IL-6 in non-steatotic and IL-10 in steatotic livers in conditions of LT and BD.

### 3.3 Origin of IL-6, IL-10, and IL-1β in non-steatotic and steatotic LT from DBDs

Since IL-6 and IL-10 might be generated in intestine and adipose tissue in the context of different liver diseases (42–44), we evaluated whether, in addition to the liver, such tissues were contributing to the increased levels of both IL-6 or IL-10 observed in non-steatotic and steatotic liver grafts, respectively. In view of that, IL-6 and IL-10 were measured in adipose tissue and intestine. Our results showed that levels of IL-6 and IL-10 in intestine and adipose tissue in recipients with either non-steatotic or steatotic grafts, respectively, were similar in all experimental groups analyzed (**Figure 6B**). Therefore, neither adipose tissue nor intestine were responsible for changes in both ILs observed in liver grafts at 4 h post-LT.

**Figure 7:**
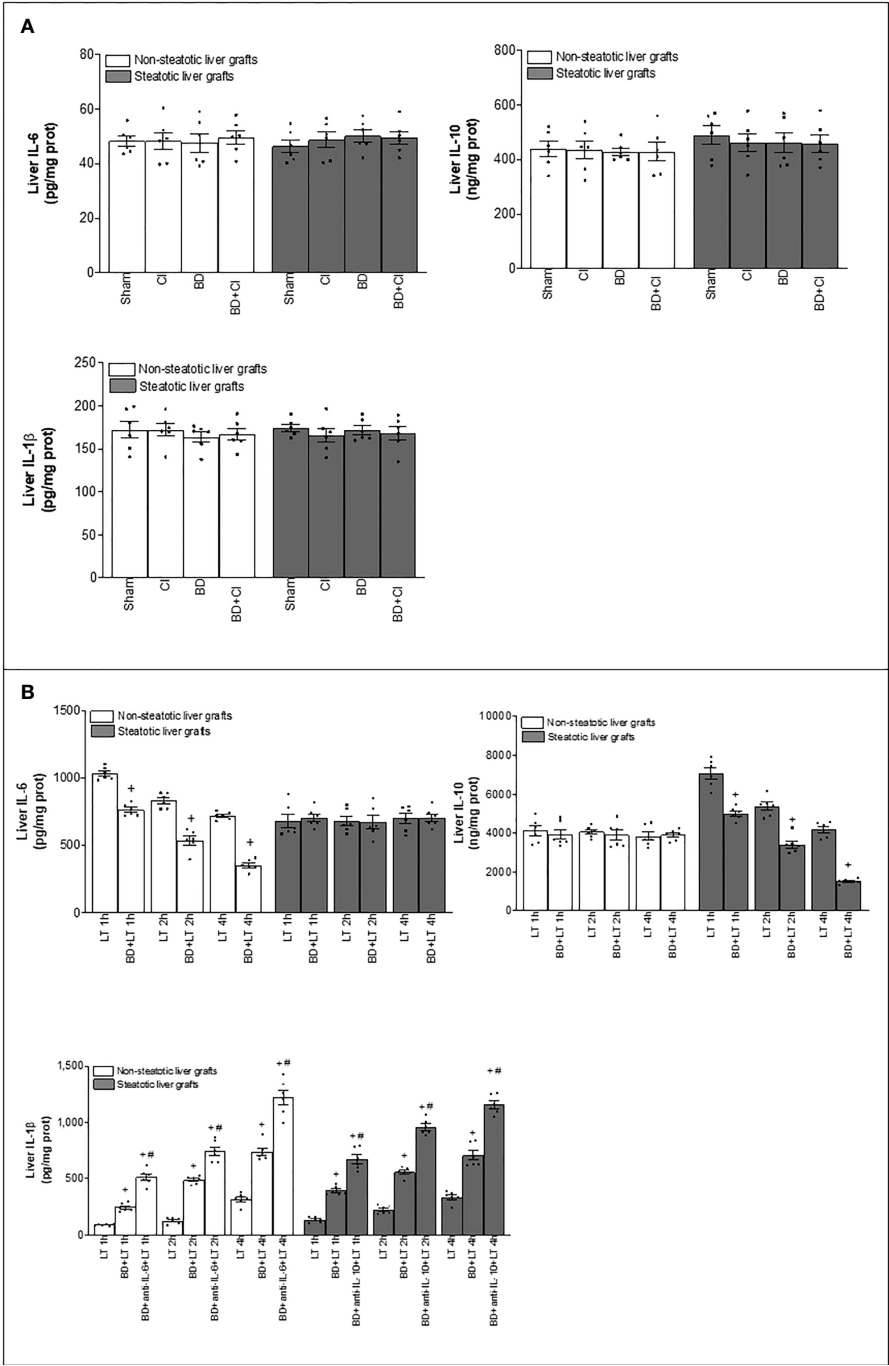
Generation of IL-6, IL-10, and IL-1β in the different stages of steatotic and non-steatotic LT from DBDs. **(A)** Hepatic levels of IL-6, IL-10, and IL-1β before implantation of liver grafts. **(B)** IL-6, IL-10, and IL-1β in liver grafts after transplantation. For A, six Ln or six Ob animals per group in each measurement; and for B, six transplants per group in each measurement. *p < 0.05 versus Sham; ^+^ *p* < 0.05 versus LT; ^#^ *p* < 0.05 versus BD+LT.

We then considered that under experimental conditions of LT and BD, IL-6 and IL-10 are generated in liver tissue. Next, we explored whether these cytokines were originated in each type of liver at graft procurement, or just after BD procedure, and if the expression of IL-1β in liver tissue (which is induced by IL-6 or IL-10 in the experimental conditions evaluated in the present study) was already altered at those points in the surgical process. Results demonstrated that the levels of IL-6, IL-10, and IL-1β were similar in Sham (without any surgical intervention), CI (livers with cold ischemia, but without BD), BD (livers with BD but without cold ischemia), and in the BD+CI group (liver grafts experiencing BD and cold ischemia) (**Figure 7A**). Thus, hepatic levels for these three cytokines increase during the reperfusion in the recipient, and this means that IL-6, IL-10, or IL-1β did not originate before the implantation of liver grafts in recipients, regardless of whether the graft comes from a BD or non-BD donor.

Finally, we evaluated whether differences in the levels of IL-1β altered by IL-6 or IL-10 were observed at earlier reperfusion times than 4 h in steatotic and non-steatotic livers. Then, liver samples were collected at 1 h and 2 h post-LT. Levels of IL-6 and IL-10 in non-steatotic and steatotic livers, respectively, gradually diminished as reperfusion occurs (**Figure 7B**). As it happened at 4 h of reperfusion, at 1 and 2 h of reperfusion, induction of BD (BD+LT) led to lower IL-6 levels in non-steatotic grafts than those observed in the LT group. In non-steatotic BD+LT groups, the higher levels of IL-6 were recorded at 1 h, whereas the lower levels were recorded at 4 h of reperfusion. On the other hand, in steatotic grafts, IL-6 levels were similar in BD+LT and LT groups at 1, 2, and 4 h of reperfusion (**Figure 7B**). In the case of IL-10, induction of BD (BD+LT) resulted in no changes for this interleukin in non-steatotic livers when compared to the LT group, at neither 1, 2, nor 4 h after reperfusion. However, when steatosis was present, a progressive reduction in the levels of IL-10 was observed as the reperfusion time proceeds. We observed reduced hepatic IL-10 in the BD+LT group, in comparison with the LT group, at 1, 2, and 4 h of reperfusion. Of interest, in BD+LT groups with steatotic livers, IL-10 was more elevated at 1 h and fewer at 4 h of reperfusion (**Figure 7B**).

Concerning IL-1β, hepatic levels were increased as reperfusion went on, and interestingly, at reperfusion times shorter than 4 h, hepatic IL-1β was higher in steatotic livers than in non-steatotic ones. In both types of liver from DBDs, IL-1β increased in the recipient at 4 h after LT (LT+BD groups), with such IL-1β levels being similar to steatotic and non-steatotic livers (**Figure 7B**). Treatment with antibodies against IL-6 (BD+anti-IL-6+LT) in non-steatotic livers, and against IL-10 (BD+anti-IL-10+LT) in steatotic grafts, augmented IL-1β even more compared to those levels from its respective BD+LT group, and levels were similar in the absence or presence of steatosis, at that reperfusion time (4 h post-LT) (**Figure 7B**). At 2 h after LT, levels of IL-1β were also higher in the BD+LT than in the LT group for non-steatotic and steatotic livers, but hepatic IL-1β in experimental groups with steatotic livers was more elevated than that recorded in respective analogous groups with non-steatotic livers (**Figure 7B**). In line with the above, when IL-6 in non-steatotic livers and IL-10 in steatotic livers were inhibited at 2 h post-LT, IL-1β levels increased in the BD+anti-IL-6+LT in grafts without steatosis and in the BD+anti-IL-10+LT group in grafts with steatosis, compared with their respective BD+LT groups. Similar to what occurred in groups without cytokine modulation, the levels of IL-1β recorded were higher in the group with steatotic livers than in non-steatotic ones (**Figure 7B**). Finally, when evaluating the hepatic levels of IL-1β in experimental groups at 1 h of reperfusion, the same pattern as observed at 2 and 4 h was registered between BD+LT and LT in both types of graft, as well as in BD+anti-IL-6+LT vs. BD+LT in non-steatotic livers and in the case of BD+anti-IL-10+LT vs. BD+LT in steatotic livers. Once again, in all the experimental groups analyzed at 1 h of reperfusion, the levels were higher in the presence of hepatic steatosis (**Figure 7B**). Contrary to what was observed during the reperfusion period for IL-6 and IL-10, the lowest levels of IL-1β in both types of grafts were recorded at 1 h of reperfusion, while the highest levels were observed at 4 h post-LT. These results demonstrated that IL-1β in non-steatotic and steatotic liver grafts is already being regulated by IL-6 and IL-10, respectively, at 1 h post-reperfusion. Remarkably, unlike what occurred in non-steatotic livers, the damaging effects of IL-1β are more intense as early as 1 h in steatotic livers, since IL-1β levels are higher in this type of liver at the start of reperfusion. This could be related to the exacerbated damage shown by steatotic livers in the conditions evaluated in the present study.

In different experimental groups, IL-1 receptor antagonist is administered to the donor since the liver graft obtained is the one that must be prepared to abolish high levels of IL-1β that will be generated later at reperfusion time. If the antagonist is administered to the recipient prior to transplantation, taking out the recipient’s liver would remove a significant amount of the IL-1 receptor antagonist. Then, after implanting the donor-derived graft, the effects of the IL-1β inhibitor would no longer be present in the recipient at concentrations adequate to eliminate the effects of IL-β that will be generated rapidly at the beginning of reperfusion in the liver graft obtained from the donor. On the other hand, our preliminary control experiments demonstrated that when IL-1 receptor antagonist administration was performed in the recipient just after reperfusion, no effect was observed (data not shown). Then, it seems that this antagonist needs more than 4 h to exert its activity, and in consequence, when we administered IL-1 receptor antagonist immediately after reperfusion, no effect was demonstrated at the time of 4 h when the sacrifice is performed. Moreover, we tried to take advantage of the time frame between the declaration of BD and organ retrieval to prevent the side effects of the drug in other organs of the recipient. Because of this, in LT from DBD, a pharmacological treatment that could be administered in the donor that will be beneficial to the recipient would be most appropriate, as we performed in the case of IL-1 receptor antagonist.

Similarly, recombinant IL-1β was administered just after BD was induced since our experience and preliminary control assays indicated that it was the optimal pretreatment time to eliminate the protective effects of IL-6 or IL-10, which occur during reperfusion time in liver grafts. From our preliminary control experiments, we observed that administration of IL-1β just after induction of BD in the donor induced an increase of such cytokine in liver tissue after 6 h of BD, and this was maintained after 4 h of cold ischemia. In this sense, levels of IL-1β in steatotic and non-steatotic liver of the BD group (6 h after BD induction) were similar to those of the Sham group. However, an increase in IL-1β was observed in steatotic and non-steatotic livers of the BD+IL-6+IL-1, BD+IL-10+IL-1, BD+IL-6+IL-1+CI, and BD+Il-10+IL-1b+CI groups when compared with the results of either the Sham or BD group (data not shown). In this way, steatotic and non-steatotic grafts from DBDs had high levels of IL-1β to eliminate the protective effects of IL-6 or IL-10 that will be exercised precisely in those same grafts obtained from donors at the reperfusion stage. Moreover, supplementation of IL-1β just after BD induction in donors did not affect plasma levels at 6 h since BD, as IL-1β in plasma samples from experimental groups where it was technically possible to obtain them (Sham, BD, BD+IL-6+IL-1, and BD+IL-10-IL-1) was unchanged in all groups (at Sham levels, data not shown). Since liver grafts are isolated from circulation in BD+IL-6+IL-1+CI and BD+IL-10+IL-1+CI groups, it is not possible to collect plasma samples from them. These findings would mean that when recombinant IL-1β is administered in donors just after BD, it is not present in the circulation but in liver tissue, and then it would not have deleterious effects on other organs, which would be beneficial in the clinical context of LT. As it was the rationale for IL-1β receptor antagonist, if we deliver IL-1β to the recipient prior to LT, recipient liver extraction would remove a significant part of the recombinant IL-1β, so that after implanting the donor-derived graft, the IL-1β would no longer be present in the recipient at concentrations adequate to eliminate the effects of IL-6 or IL-10 during reperfusion time. From our previous control experiments, we observed that when administering IL-1β in the recipient post-LT, an increase in IL-1β levels in the plasma from recipients of groups BD+IL-6+IL-1+LT and BD+IL-10+IL-1+LT was registered at 4 h of reperfusion (data not shown). Therefore, we tried to avoid these high plasma IL-1β levels, in order to avoid a potential negative effect of this recombinant interleukin in other tissues from the recipient, which is crucial in the clinical scenario of LT.

### 3.4 Role of NO on IL levels in LT of steatotic and non-steatotic grafts recovered from DBDs

In consideration of the previous data that suggest that the effects resulting from NO could involve signaling mechanisms requiring interleukins (12, 16, 17, 19–21), we evaluated whether endogenous NO affects IL-6, IL-10, and IL-1β in non-steatotic and steatotic grafts from DBDs.

**Figure 8:**
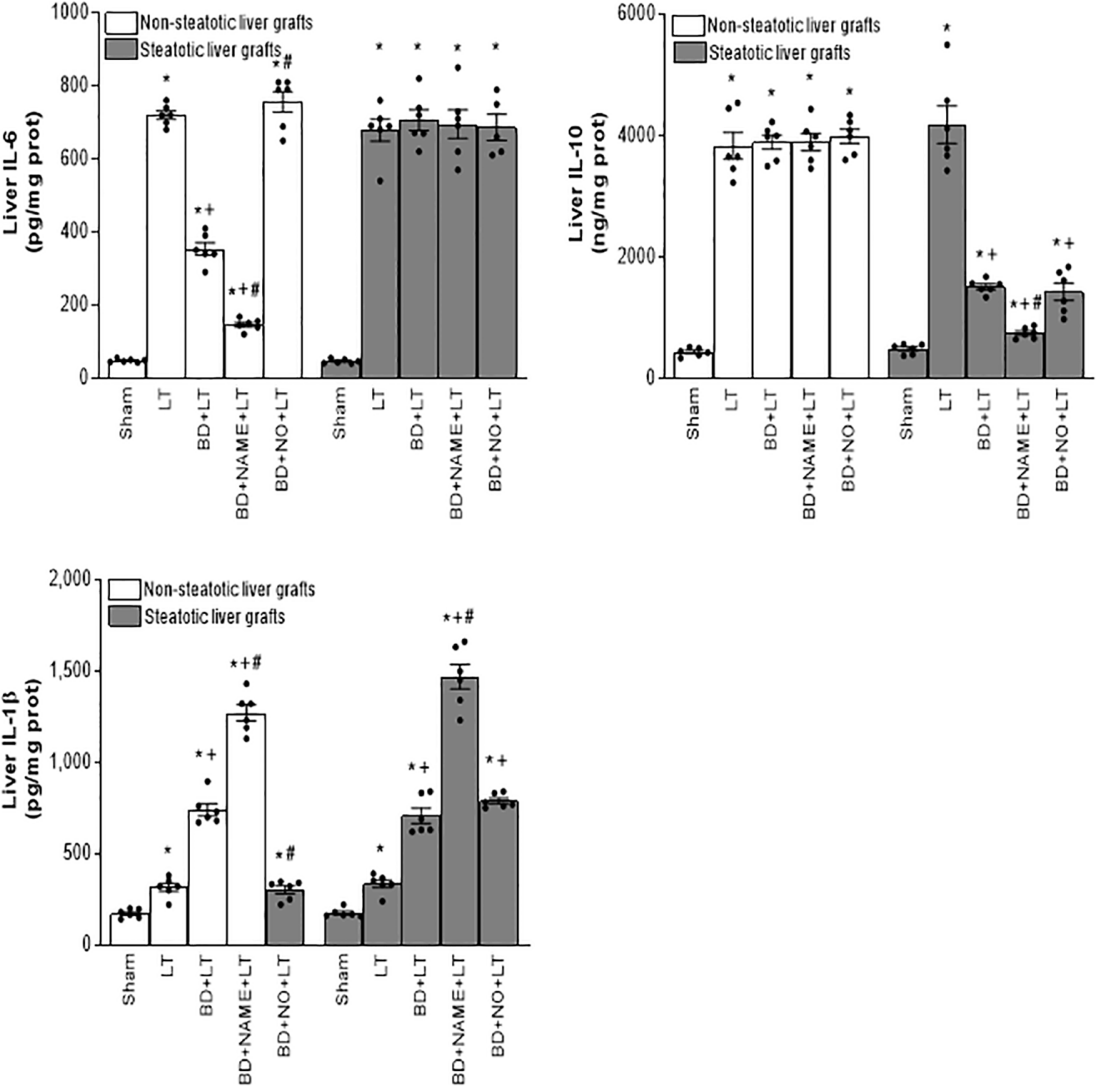
Effect of endogenous and exogenous NO on interleukin levels in steatotic and non-steatotic LT from DBDs. IL-6, IL-10, and IL-1β in liver. For A and B, six transplants per group in each measurement. *p < 0.05 versus Sham; ^+^ *p* < 0.05 versus LT; ^#^ *p* < 0.05 versus BD+LT.

**Figure 9:**
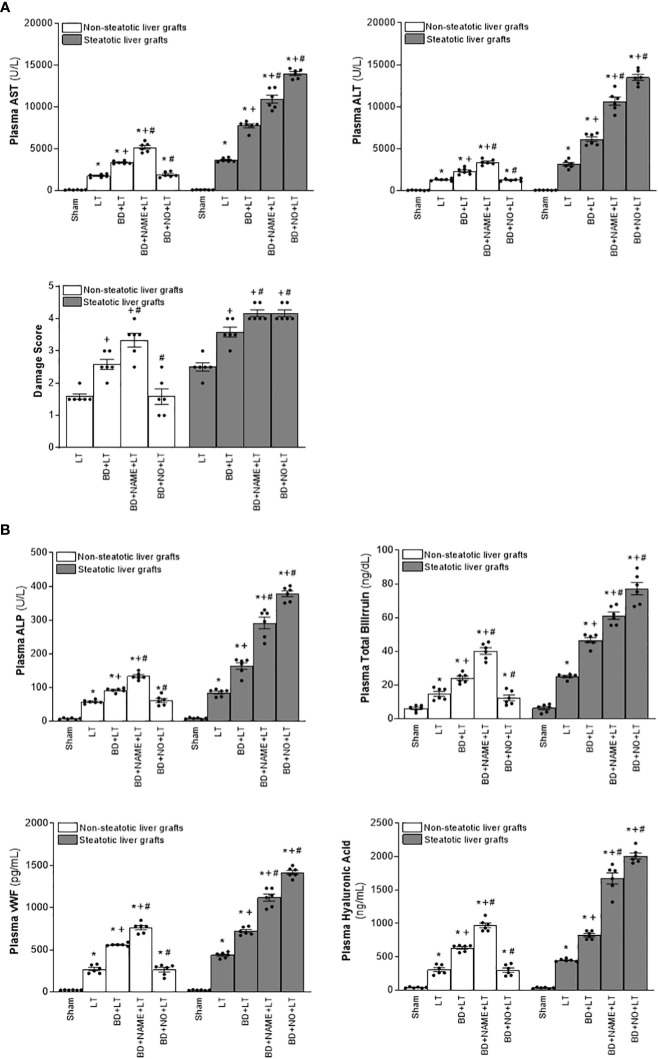
Involvement of interleukins in the effects of NO on damage in steatotic and non-steatotic LT from DBDs. **(A)** ALT and AST levels in plasma and liver damage score. **(B)** ALP, total bilirubin levels, vWF, and HA levels in plasma. For A and B, six transplants per group in each measurement. *p < 0.05 versus Sham; ^+^ *p* < 0.05 versus LT; # *p* < 0.05 versus BD+LT.

**Figure 10:**
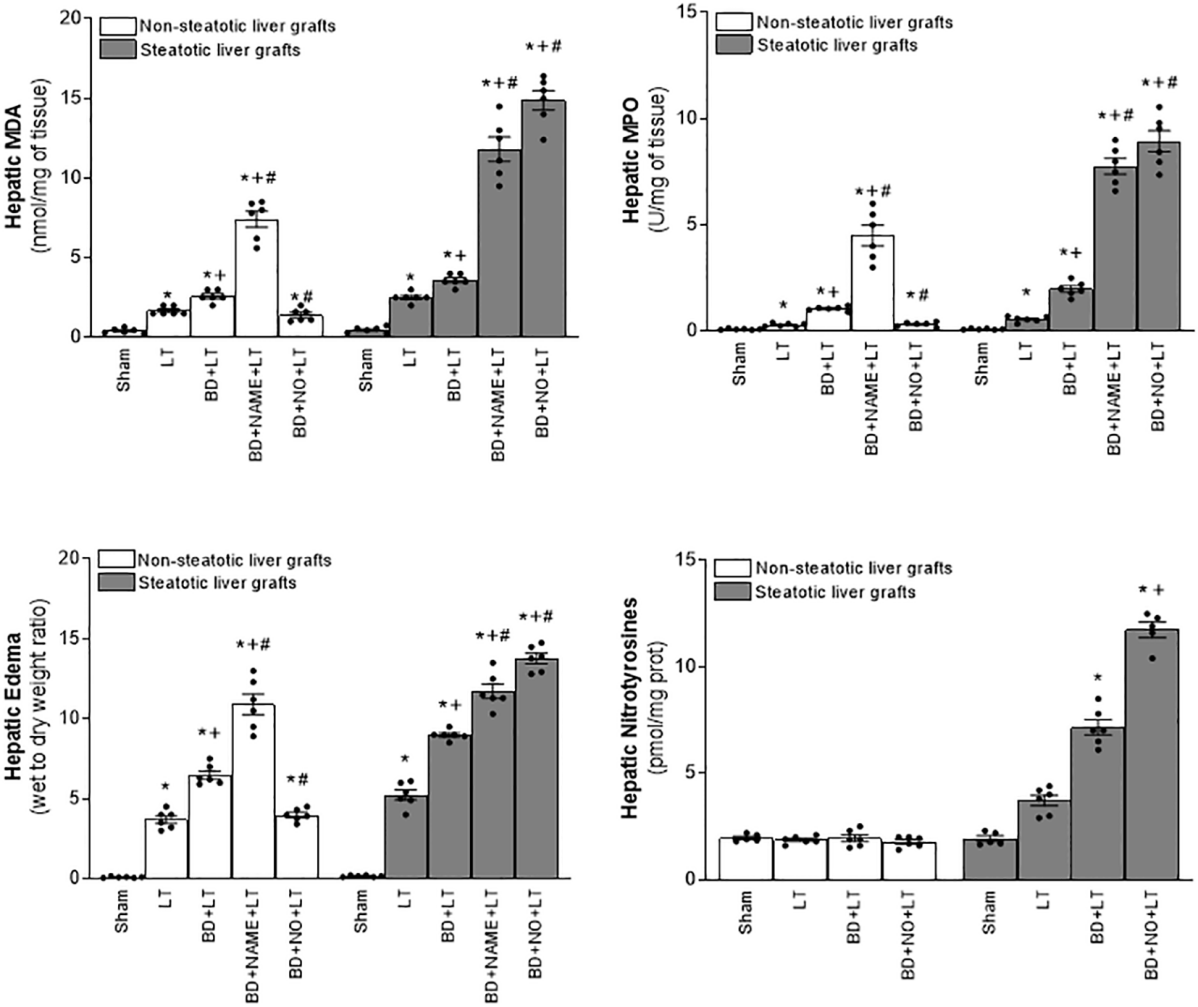
Involvement of interleukins in the effects of NO on inflammation in steatotic and non-steatotic LT from DBDs. Hepatic levels of MPO, edema, MDA, and nitrotyrosines. For A and B, six transplants per group in each measurement. *p < 0.05 versus Sham; ^+^ *p* < 0.05 versus LT; ^#^ *p* < 0.05 versus BD+LT.

In non-steatotic grafts from DBDs, NO inhibition (BD+NAME+LT) resulted in reduced IL-6 and high IL-1β (**Figure 8**), and exacerbated liver injury and inflammatory response in comparison to the experimental group BD+LT (**Figures 9**, **10**). In steatotic grafts, suppression of NO (BD+NAME+LT) reduced hepatic IL-10 and worsened hepatic damage and inflammation in comparison to the BD+LT group; all of this was associated with more elevated IL-1β levels than those in the BD+LT group (**Figures 8**–**10**).

In light of the beneficial effects of endogenous NO for hepatic grafts obtained from DBDs, we decided to assess if administration of exogenous NO would increase the benefits observed for endogenous NO. In grafts in the absence of steatosis obtained from DBDs, we observed that the administration of exogenous NO (BD+NO+LT) increased IL-6 and that this was associated with reduced IL-1β levels and protection against hepatic damage and inflammation, compared to the BD+LT group (**Figures 8**–**10**). In hepatic grafts from DBDs in which steatosis was present, the same level of administration of NO (BD+NO+LT) did not have an effect and was unable to induce changes in either IL-10 or IL-1β with respect to the experimental group BD+LT, without any treatment (**Figure 8**). In addition, NO supplementation severely aggravated all the parameters of hepatic damage and inflammation more than in the BD+LT group (**Figures 9**; **10**). The effect induced by NO supplementation in steatotic grafts was related to an excess in nitrotyrosine generation, indicative of an increase of oxidative stress involving peroxynitrite (ONOO^−^) formation, a highly oxidizing and cytotoxic reactive species. Thus, nitrotyrosine levels were notably increased in the experimental group BD+NO+LT, in comparison with the BD+LT group, for liver grafts in which steatosis was present; however, this did not occur with the same treatment in non-steatotic grafts from DBDs (**Figure 10**).

**Figure 11:**
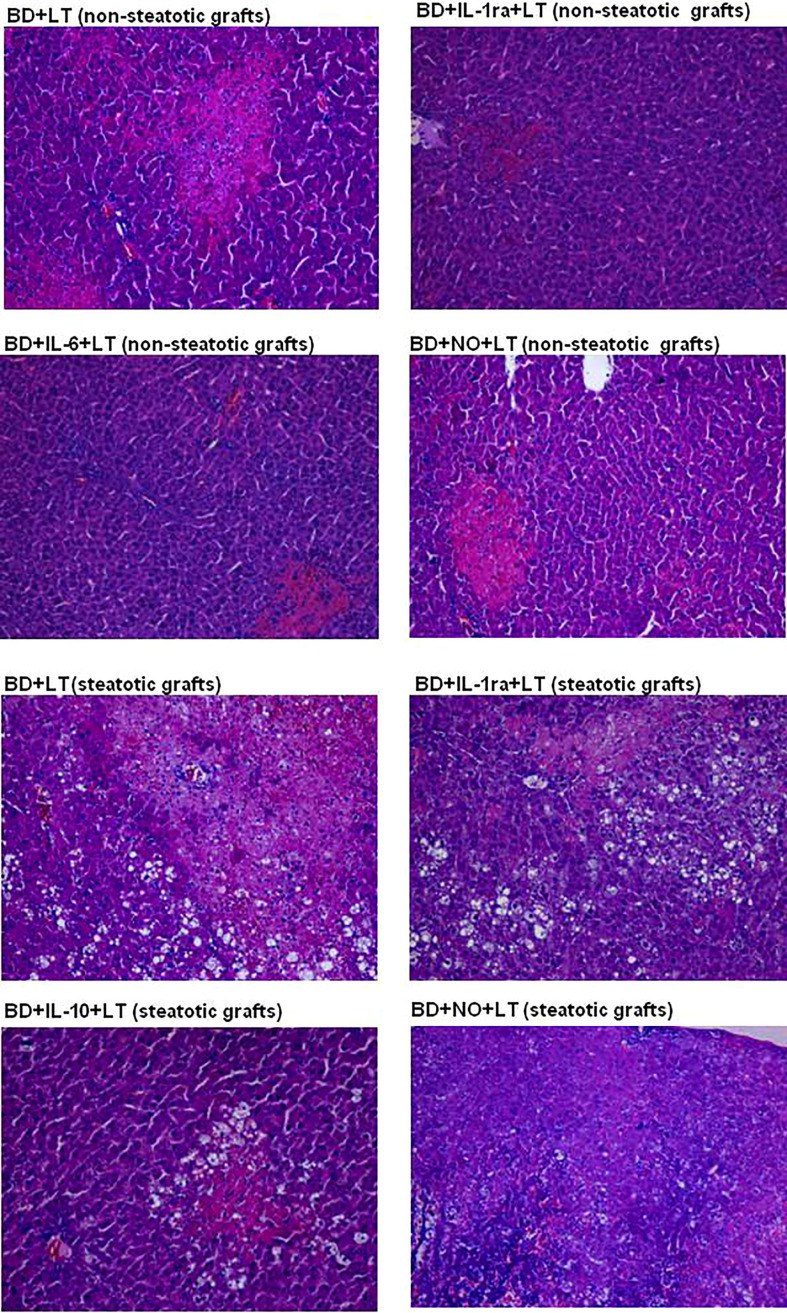
Representative photographs of histological changes in non-steatotic livers and steatotic LT from DBDs. In the BD+LT group, histological evaluation of non-steatotic livers showed moderate multifocal areas of coagulative necrosis and neutrophil infiltration, randomly distributed throughout the parenchyma, whereas the extent and the number of necrotic areas were reduced in the BD+IL-1ra+LT, BD+IL-6+LT, and BD+NO+LT groups (4×). Steatotic liver grafts from the BD+LT and BD+NO+LT groups showed extensive and confluent areas of coagulative necrosis, while the extent and the number of necrotic areas were reduced in the BD+IL-1ra+LT and BD+IL-10+LT groups (4×).

In **Figure 11**, we show representative histological images from the different interventions performed in the study aimed at protecting steatotic and non-steatotic liver grafts from DBDs. Our histological assessment of non-steatotic livers in the BD+LT group revealed a moderate presence of multifocal areas of coagulative necrosis and neutrophil infiltration, whose distribution across the parenchyma appeared to be random. In contrast to this, the necrotic areas in non-steatotic grafts from the experimental groups BD+IL-1ra+LT, BD+IL-6+LT, and BD+NO+LT were observed to be reduced in extent and number. In steatotic liver grafts from the BD+LT and BD+NO+LT groups, we observed areas with coagulative necrosis that were confluent, extensive, and severe. In contrast, there were fewer necrotic areas whose extent was reduced in grafts in which steatosis was present from the BD+IL-1ra+LT and BD+IL-10+LT groups.

## 4 Discussion

Herein, we show new endogenous signaling pathways in LT from DBDs, namely, IL-6/IL-1β in non-steatotic liver grafts and IL-10/L-1β in steatotic ones; these different mechanisms that depend on the presence of steatosis had not been reported to date. Our results indicated that when endogenous IL-6 in non-steatotic livers and IL-10 in steatotic livers, always from DBDs, are pharmacologically inhibited, then IL-1β is increased and the damage and inflammation are exacerbated. Thus, endogenous IL-6 (for non-steatotic livers) and endogenous IL-10 (for steatotic ones) exert benefits through IL-1β inhibition, which, in turn, lessen cAMP generation. The suppression of both mediators (IL-1β and cAMP) downstream IL-6 or IL-10 is responsible for the protective effects of these cytokines. In addition, present research evidenced that NO is upstream of the endogenous IL-6 or IL-10 in the setting of LT from DBDs, thus defining the following endogenous signaling pathways: NO/IL-6/IL-1β in non-steatotic grafts from DBDs, and NO/IL-10/IL-1β for steatotic grafts from DBDs. Inhibition of NO synthesis abrogated the benefits of endogenous IL-6 and IL-10 in non-steatotic and steatotic grafts, respectively, consequently increasing hepatic IL-1β and cAMP, and exacerbating inflammation and damage, more than BD did by itself in LT.

In contrast to studies in the literature that report that there is a possible benefit of both exogenous IL-10 (15) and IL-6 (19) in both liver types under warm ischemia conditions, the results of the current study in LT from DBDs indicate the specificity of IL-6 and IL-10 to protect each type of the liver. Such differential effects of IL-6 and IL-10 are not surprising considering the huge amount of evidence concerning different signaling pathways underlying injuries of the type I/R in liver that are either steatotic or non-steatotic (62–65). In addition, the type of surgical procedure (warm I/R versus LT from DBDs) should be taken into account, since the pathogenic mechanisms underlying warm I/R and LT from DBDs are quite different (26, 66). In the same way, different ischemia times (60 min in warm I/R versus 4 h in LT from DBDs) and the presence or absence of BD, among other aspects, are known to distinctly affect the underlying signaling pathways of hepatic damage (67–69).

In the present study, hepatic IL-1β levels seem to be similar in LT groups of non-steatotic and steatotic grafts, while liver damage was higher in groups with steatotic grafts. Although these results might seem incongruous, it should be noted that IL-1β levels in liver tissue do not determine the contribution to liver injury and/or the vulnerability of steatotic livers subjected to transplantation. In the present study, the participation of IL-1β in the exacerbated liver damage observed in steatotic livers is demonstrated from the evidence that we next describe. First of all, the involvement of a mediator in liver damage is evidenced by pharmacologically modulating such mediator and verifying if the liver damage parameters are altered, as we have previously demonstrated in numerous research works from our group (48, 68, 70–72). In this sense, the present study investigated the role of IL-1β in the damaging effects induced by BD in both steatotic and non-steatotic liver grafts. Thus, treatment with an IL-1 receptor antagonist (BD+IL-1ra+LT) attenuated damage in both types of liver since when compared to the group without that intervention (BD+LT), all parameters for hepatic injury and inflammatory response were reduced. Thus, we observed that inhibition of IL-1β action reduced injury both in the presence and in the absence of steatosis, which means that IL-1β is involved in hepatic damage induced by BD and LT, in both types of grafts. In the case of steatotic grafts, inhibition of IL-1β resulted in a greater degree of reduction in liver damage and inflammation compared to the magnitude of reduction seen in non-steatotic livers. Then, our results show that IL-1β is responsible for the exacerbated damage observed in steatotic grafts when they are subjected to BD and LT, since when the action of this cytokine is inhibited, the higher liver damage recorded in the BD+LT group with steatotic grafts is absent. In the present study, the effect of the IL-1β antagonist in the LT groups without BD was not analyzed, since the investigation is focused on the surgical context of BD+LT, which are the most approximate conditions to what is presented in clinical practice. Researching these two combined experimental settings could yield the best results, which are of translational interest. Secondly, in the context of liver damage associated with transplantation, it has been reported that equal levels of a mediator, for instance when administering NO or cortisol at the same dose, can cause different effects on hepatic injury in steatotic and non-steatotic livers (2, 48). In the present research, similarly to that occurring with IL-1β at 4 h after transplantation, supplying a NO donor at the same dose in both liver types led to different results: NO donor was beneficial in non-steatotic livers but resulted in detrimental effects in the presence of steatosis. Indeed, the steatotic livers show more vulnerability to the generation of peroxinitrites in the presence of NO donors than non-steatotic ones. Therefore, considering the above and the fact that steatotic livers are considered pathological (73), it is not surprising that at the same hepatic levels of IL-1β, the damaging effects of this cytokine may be more evident in steatotic livers, than in the case of normal livers. This is not unexpected owing to the widely documented differences that exist between steatotic and non-steatotic livers, with respect to molecular mechanisms, and morphological and functional differences underlying I/R liver injury, which inevitably occurs when both types of grafts undergo transplantation (74, 75). Additionally, it is important to mention that in the surgical setting of LT and BD, signaling mechanisms downstream IL-1β involved different levels of cAMP depending on the type of liver, and this occurred even though both steatotic and non-steatotic livers have similar levels of IL-1β. Our results indicated that levels of cAMP were higher in experimental groups with steatotic livers than in those with non-steatotic ones submitted to LT and BD, and it was demonstrated (through an experiment involving inhibition of IL-1β action) that IL-1β was responsible for cAMP generation in such experimental conditions. Noticeably, hepatic levels of cAMP induced by IL-1β was in parallel with degree of liver damage observed in each type of graft, that is, higher cAMP levels in steatotic livers subjected to LT and BD, compared to the levels recorded in the homologous group with non-steatotic livers. This agrees with a previous study evaluating the role of cAMP in LT from donors without BD, which indicated that increases in cAMP observed in steatotic grafts were responsible for the vulnerability of steatotic livers to I/R and exacerbated damage (76). Finally, we also explored levels of IL-1β at earlier reperfusion times than 4 h in steatotic and non-steatotic livers. We found that IL-1β in liver increased as reperfusion occurs and such enhancement was more apparent in the presence of steatosis. After 1 and 2 h after LT, levels of IL-1β increased in livers with and without steatosis when undergoing LT and BD, but hepatic IL-1β was more elevated in steatotic livers than those recorded in respective analogous groups with non-steatotic livers. These results indicate that, unlike what occurred in non-steatotic livers, the damaging effects of IL-1β are more intense as early as 1 h in steatotic livers, thus reinforcing the conclusion that IL-1β contributes to the exacerbated damage shown by steatotic livers in the conditions evaluated in the present study.

We postulated that the mechanism of action of IL-6 or IL-10 in non-steatotic and steatotic livers, respectively, is the inhibition of IL-1β production, which, in turn, downregulates cAMP. Abolition of IL-1β was crucial to achieve benefits of IL-6 or IL-10 against liver injury. Several findings that support the existence of this signaling mechanism are further revealed. In non-steatotic livers undergoing LT and BD, we found that if IL-6 is inhibited (BD+anti-IL-6+LT), hepatic levels of IL-1β are increased only in non-steatotic livers and liver damage is exacerbated in this type of liver. All of this means that in LT and BD in non-steatotic livers, IL-6 reduces IL-1β, which is necessary to limit liver injury. If IL-6 is inhibited but the action of IL-1β is also inhibited, there is no aggravated liver damage. On the other hand, administration of exogenous IL-6 (BD+IL-6+LT group) reduced damage parameters and decreased hepatic IL-1β in non-steatotic liver. Benefits from IL-6 were abolished if we administered IL-1β. This demonstrates that IL-1β is a downstream mediator of IL-6 and its regulation is a fundamental part of the signaling mechanisms underlying beneficial effects of IL-6 in non-steatotic grafts subjected to LT and BD. In the case of steatotic livers subjected to LT and BD, suppression of IL-10 (BD+anti-IL-10+LT) increased damage, inflammation, and IL-1β in liver. Such effects were not observed when both IL-10 and IL-1β effects were repressed. Therefore, in steatotic liver grafts from LT and BD, IL-10 decreases IL-1β levels, and this is required to restrain hepatic damage. When IL-10 is hindered, IL-1β increases and injury is impaired in steatotic livers. Provided that IL-10 is inhibited but also prevents IL-1β action, then intensified injury is absent in that type of liver. Treatment steatotic liver grafts with exogenous IL-10 reduced injury and inflammation and decreased IL-1β levels in that type of liver. These advantageous effects in steatotic livers were abolished if we administered IL-1β. Hereby, IL-1β is a downstream mediator for IL-10 and its regulation is crucial as it is part of the signaling pathway necessary to accomplish protection from IL-10 in steatotic grafts subjected to LT and BD. Last, we explore whether cAMP could be a mediator downstream IL-1β. In this sense, we observed that (a) cAMP levels increased in both steatotic and non-steatotic grafts from DBDs and transplanted, and (b) in conditions of pharmacological modulation of IL-1β, cAMP levels were not affected. These findings confirmed that, downstream of IL-6 in normal livers and IL-10 in steatotic livers, cAMP is a mediator of the deleterious effects of IL-β. Interestingly, hepatic cAMP levels reflected the different degrees of injury seen between steatotic and non-steatotic livers subjected to the same surgical conditions evaluated in the present study. Accordingly, previous reports described that cAMP affected the main mechanisms responsible for the vulnerability of steatotic livers to I/R damage (without BD), including oxidative stress, endothelial cell damage, and edema (45). Hence, in our hands, cAMP also appears to be related to the exacerbated damage suffered by livers with steatosis in LT and BD, although more experiments need to be performed to verify this.

The present investigation suggests that in LT of grafts from DBDs, the source of IL-6, IL-10, and IL-1β is the liver itself, without involving other organs as a source of these cytokines. Hepatic levels for these three cytokines were modified during the reperfusion in the recipient, and none of them suffered changes either in the donor with BD or during back table surgical procedures (before the implantation of liver grafts in recipients). In fact, our results indicated that after transplantation in recipients from DBDs, IL-6 and IL-10 in non-steatotic and steatotic livers, respectively, gradually diminished as reperfusion occurs. Contrary to this, IL-1β in such both liver types increased as reperfusion progressed. All of this demonstrated that IL-1β in non-steatotic and steatotic liver grafts is already being regulated by IL-6 and IL-10, respectively, at early reperfusion times. Then, during reperfusion in LT from DBDs as IL-6 or IL-10 decreases in each type of liver, the production of IL-1β also increases. Remarkably, unlike what occurred in non-steatotic livers, the damaging effects of IL-1β were more intense as early as 1 h in steatotic livers, since IL-1β levels are higher in this type of liver beginning reperfusion. As previously mentioned, this could be related to exacerbated damage shown by steatotic livers in the conditions evaluated in the present study.

Several investigations have found in murine and cell-based studies that IL-6 signaling for more than 24 h induces insulin resistance in adipose and hepatic tissue (77–79). Similarly, clinical and experimental studies have also shown that IL-10 is directly related to insulin action, *in vivo*, and that exogenous IL-10 could improve insulin action in skeletal muscle and liver (80–82). In our hands, treatment with either IL-6 or IL-10 did not induce changes in plasma levels of glucose, insulin, or lipid profile parameters (including HDL, LDL, triglycerides, and cholesterol) from recipients transplanted after 4 h of reperfusion, when compared with animals without treatment (that is, BD+IL-6+LT and BD+IL-10+LT vs. BD+LT) with steatotic and non-steatotic liver grafts from DBDs (data not shown). Therefore, modulation of either IL-6 or IL-10 does not have effects on the metabolic profile of the recipients under the conditions evaluated in the present study, the early period of reperfusion. Such opposite results concerning literature reports (77–82) are not surprising considering the facts described below. One of the cited studies used a model of 3T3-L1 adipocytes or isolated human fat cells (79). It is widely known that experimental models based on isolated cell cultures do not reproduce the clinical setting of LT from DBDs (in which the response of a single cell type is not observed but rather, a response produced by the complex interrelationship of various cell types). Therefore, it is foreseeable that the results obtained in the study of a single isolated cell type will not be reproduced in the *in vivo* experimental conditions of our study. In another cited study, IL-6 was administered continuously in a mouse model without any liver injury, and biochemical features of insulin resistance were observed immediately after cessation of IL-6 supplementation for 5 to 7 days (78). In our experimental conditions, the administration of IL-6 was performed only twice, 24 h and 12 h before the induction of BD, which means that IL-6 would affect livers experiencing the detrimental effects of BD and I/R injury associated with transplantation. In this latter experimental setting, IL-6 had no effect on insulin resistance. All this points to the fact that the effect of IL-6 on insulin resistance and metabolic profile could depend on liver pathology as well as the different experimental conditions. This is not surprising due to the widely documented differences that exist between livers undergoing I/R and those that are not subjected to this type of damage, with respect to molecular signaling and functional differences (83, 84). Regarding IL-10, in a study with mice presenting severe hepatic steatosis and defective insulin signal transduction plus diabetes, animals were treated with two daily doses of an IL-10 inhibitor for 5 days. Upon such time, mice exhibited worsening of insulin signaling and the activation of gluconeogenic and lipidogenic pathways, suggesting that IL-10 exerts a protective role for liver insulin resistance associated with steatosis (82). Under the conditions evaluated in our study, the administration protocol to modulate the action of IL-10 was different and shorter, in addition to the fact that although animals with steatotic livers were used in some of our experimental groups, they did not present diabetes and instead were subjected to the injurious consequences of BD and cold ischemia. These marked differences in the experimental settings could explain why, in our investigation, IL-10 has no effect on insulin resistance at 4 h post-transplantation. On the other hand, insulin resistance and alterations in adipokines such as adiponectin and leptin might have a central role in liver metabolic damage after transplantation, as stated in a recent clinical study by Eshraghian et al. (85). Regulation of adipocytokines has been shown as a promising strategy for reducing I/R injury in steatotic livers, based on adipocytokines’ effects on inflammation, steatosis, fibrosis, and molecular pathways of liver damage and regeneration (86). However, a role for adipokines and insulin resistance in pathogenesis of steatosis does not occur at least in the conditions evaluated in our study, since levels of glucose, insulin, and adiponectin were similar in plasma at 4 h of reperfusion in experimental groups BD+IL-6+LT, BD+IL-10+LT, BD+anti-IL-6+LT, BD+anti-IL-10+LT, and BD+LT, with steatotic and non-steatotic liver grafts from DBDs (data not shown). Noticeable differences between the clinical study of Eshraghian et al. and ours might account to explain such results. We evaluated biochemical parameters at 4 h after transplantation, as usually performed in investigations evaluating injurious effects of hepatic I/R inherent with liver surgery; meanwhile, in the clinical study, alterations were evaluated at a mean time of 38 months after LT. Also, in our experimental model, all recipients of LT were lean animals without any pathology, whereas recipients in Eshraghian et al.’s research had diverse liver pathologies before undergoing transplantation.

The mechanisms through which IL-6 and IL-10 modulate the generation of IL-1β were not part of the objectives of the present study. On the other hand, the possibility that IL-6 or IL-10 influenced Kupffer cells and infiltrated recipient-derived macrophages after transplantation should not be discarded. In fact, according to data reported in the literature, IL-6 and IL-10 are strongly related to Kupffer cells’ action (16, 87–89). It has been reported that IL-10 may regulate proinflammatory mediators’ release in a liver perfusion model or in isolated Kupffer cells (89) and that IL-10 via Kupffer cells protected steatotic livers modulating the production of IL-1β in a mouse model of warm ischemia (16). Because of this relation between IL-6 and IL-10 and Kupffer cells in such surgical conditions, the involvement of the regulation of Kupffer cells by IL-6 and IL-10 in the experimental model presented in this research could not be discarded. Thus, elucidating the participation of Kupffer cells as a mediator through which IL-6 or IL-10 induces IL-1β production might deserve future intensive investigations.

Results presented in the present manuscript indicate that both IL-6 and IL-10, through the regulation of IL-1β, affect infiltration and accumulation of neutrophils. This conclusion was reached, since MPO levels were affected when the cytokines IL-6, IL-10, and IL-1β were pharmacologically modulated, either separately or in combination (depending on the type of livers in which they were shown to exert their action). Additionally, there are many reports in the literature indicating that IL-6 and/or IL-10 are capable of also modulating macrophages and chemokines (90–98), and therefore, the possibility that this is also happening under the conditions of the present study cannot be dismissed. IL-6 and IL-10 have been identified as important modulators of macrophage activity in the context of liver injury and disease (91–98). Under similar conditions of hepatic injury, IL-10 inhibited production of various CXC chemokines (90, 92), and in a rat model of orthotropic LT, IL-10 modulated CXCL2 chemokine or macrophage activity, thus affecting the early period after I/R (91). Regarding IL-6, it upregulated CXCL1 chemokine in conditions of injury in several tissues including liver (93, 94), and in experimental models of hepatic I/R injury, CXCL10 and IL-6 were strongly correlated (96). Also, IL-6 would be related to the activity and ligands of CXCL2/CXCR2 chemokine in partial hepatectomy and I/R (95). Interestingly, a correlation between CXCL10 and IL-6 has been recently reported in human subjects after solid organ transplantation with BD donors (97). Thus, it would be valuable to explore issues related to chemokines and macrophages in future research that are currently outside the scope of this manuscript.

Other immune cells such as natural killer (NK) cells, dendritic cells (DCs), T lymphocytes (LT) CD4+ and CD+8, or Kupffer cells (KC) seem to have an important role in mechanisms underlying injurious postoperative outcomes in the setting of LT (99), and thus, a relationship between signaling pathways described in the present research (IL-6/IL-1b or IL-10/IL-1b) and such immune cells, in the context of LT from DBDs, should not be discarded. NK cells have been reported as modulators of I/R injury and, according to different authors, the infiltration of these immune cells into the liver exacerbates liver injury, promoting other inflammatory cells’ infiltration in the graft (100). A relation has been established between NK cells and IL-6 or IL-10. IL-6 prevents liver inflammation via suppression of NK cells, since administration of IL-6 markedly attenuated the ability of NK cells to kill hepatocytes *in vitro*, which has been suggested to play an important role in the pathogenesis of hepatitis (101). In addition, IL-10 has been reported as an inhibitor of NK cells and their receptors, downregulating its cytotoxic activity in LT (100). Recently, compelling evidence has delineated the role of DCs in hepatic I/R injury and several results support that DCs modulate levels of IL-6 and IL-10 in liver submitted to ischemia injury (102, 103). T lymphocytes (CD4+) produce chemokines that amplify KC activation, which promote neutrophil recruitment and adherence into the liver sinusoids, aggravating IR injury (104). CD4+ T cells accumulate rapidly in mouse LT following cold storage, and in this sense, as early as at 1 h post-transplant, a massive infiltration of liver graft with CD4 T cells is found. In such conditions, CD4+ T cells express TIM-1 and modulation of this signaling mediator has resulted in the regulation of IL-6 and IL-10 production, and amelioration of cold hepatic ischemia-mediated LT inflammation and damage (105). After I/R injury, steatotic livers have showed increased infiltrating CD8+ cells in association with high levels of parameters of liver damage and proinflammatory cytokines (106). As occurring in the case of CD4+ cells, some reports have indicated that CD8+ cells may also regulate IL-6 and IL-10 in liver tissue (107, 108). Interestingly, induction of IL-6 expression in the liver prevented CD8+ T cell-mediated liver injury (109). All this constitutes interesting topics to be addressed in years to come, in order to better comprehend multiple complex interrelations between immune cells and inflammatory mediators that are established in liver grafts suffering from I/R inherent with LT and injurious effects of BD.

The data provided in this study determined that IL-6/IL-10 protects liver grafts from DBDs and, thus, hepatocytes, in an indirect way, if considering that the benefits of IL-6/IL-10 can only be achieved through the regulation of IL-1β. The mechanisms triggered downstream of the NO/IL-6/IL-1β pathway in non-steatotic livers and of the NO/IL-10/IL-1β pathway in steatotic ones resulted in beneficial effects such as reduction of endothelial damage, neutrophil accumulation, oxidative stress, and cellular edema. Surely, the attenuation of these cellular events protects the integrity of hepatocytes, and indeed, the detrimental effects of endothelial damage, neutrophil accumulation, and oxidative stress on hepatocytes are well known (62, 86, 110). In this sense, the reduction of such parameters was precisely associated with a decrease in graft damage parameters directly related to hepatocytes, such as ALT levels (released from hepatocytes) and damage score (evaluated directly in a liver tissue sample in which it is possible to observe the integrity or destruction of hepatocytes). Some studies have indicated that both IL-6 (in a rat model of partial LT) and IL-10 (in a rat model of hepatic I/R injury) have beneficial effects on hepatocytes, since they demonstrated that these cytokines promoted the proliferation of hepatocytes (111, 112). In these terms, there is the possibility that in the model of the present study (liver transplant with grafts from DBDs) IL-6 and/or IL-10 could also exert a direct action on the functionality and integrity of the hepatocyte. On the other hand, it is important to mention that to determine a direct hepatocyte response to IL-6 or IL-10 modulation, evaluation in an *in vitro* model is required, to isolate hepatocytes from the influence of non-parenchymal liver cells. Therefore, in the *in vivo* model used in the present investigation, it is not possible to conclude whether a cell type is being directly affected by IL-6 or IL-10, since the damage and inflammation parameters obtained from the LT from the DBD model are the result of the interaction of all cell types present in the liver. Although some aspects of I/R inherent to LT have been replicated *in vitro* to determine direct effects on hepatocytes (113–115), they are by no means the same conditions that occur when LT is performed in clinical practice. The great relevance of *in vivo* models is that they allow mimicking what occurs in clinical settings, where an interaction between different cell types occurs, and not the effects of a single cell type. Our study is an investigation of translational medical science, and in this field, what is sought is to reproduce as faithfully as possible in an experimental model what happens in clinical practice. This is extremely important so that in the short or medium term, the results can have an application in the clinical setting (116, 117) and, in that way, contribute to solving problems in LT from DBD that arises in our investigation.

Some studies in myocardial ischemia (24) and warm hepatic I/R (15) indicate that NO can induce IL-10 production, and in the setting of partial LT, NO has been shown to have the capacity to regulate generation of IL-6 (25). Moreover, I/R experimental models have also previously been used to demonstrate that NO inhibits the production of IL-1 (15), as is also the case when LPS induces hepatotoxicity (118). As data from the present research pointed to NO being upstream of IL-6 or IL-10, we investigated whether exogenous NO administration could confer protection against injury in LT from DBDs by stimulating IL-6 release in non-steatotic livers and IL-10 in steatotic ones. In our hands, in non-steatotic grafts from DBDs, exogenous NO increased IL-6, and this reduced IL-1β, thereby protecting against oxidative stress, inflammation, and damage. However, treatment with exogenous NO did not modify the levels of either IL-10 or IL-1β in steatotic grafts from DBDs, but worsened oxidative stress, inflammation, and damage.

It is known that NO can act as an antioxidant, a vasodilator, and an antineutrophil, which means that this molecule has important potential to provide protection (48, 119). In contrast, it is also known that when combined with superoxide (O_2_^−^), the same NO molecule can form the detrimental ONOO^−^ ion (120). If *in vivo* production of NO as well as O_2_^−^ are at high levels, then ONOO^−^ may be formed, with the well-known concomitant oxidative and cytotoxic effects (48, 121, 122). Antioxidant defenses in cells are reduced by ONOO^−^, and it can also inactivate certain enzymes and in some proteins lead to nitration of tyrosine residues, which can have negative effects on different functions and also interfere with the process of signal transduction (123). Also, ONOO^−^ promotes an inflammatory response in the liver, which includes, among other cellular events, the accumulation of neutrophils and cellular edema (124, 125). Interestingly, our results concerning nitrotyrosine hepatic levels indicate that ONOO^−^ is potentially one of the most important reactive oxidants when we are considering steatotic liver grafts from DBDs. In agreement with this finding, we also found that when dealing with steatotic liver grafts obtained from DBDs, an increase in the levels of nitrotyrosine was also related to the negative effects that substances that are exogenous donors of NO had in terms of liver injury, inflammation, and levels of oxidative stress. This could partly explain why exogenous NO is harmful in the surgical setting of steatotic LT from DBDs. In contrast, in non-steatotic liver grafts from DBDs, exogenous NO (which was not associated with nitrotyrosine generation) protected against damage, oxidative stress, and inflammation. As previously mentioned, ONOO^−^ is formed when the condition of high production of both NO and O_2_^−^ occurs. In steatotic grafts from DBDs, these conditions come about when exogenous NO is administered, since these grafts generate reactive species intensely in LT from DBDs, and therefore, peroxynitrites are formed and liver damage is exacerbated. On the other hand, in the case of non-steatotic grafts from DBDs, since far fewer ROS are produced in LT from DBDs, when they are treated with exogenous NO, the conditions necessary to form ONOO^−^ do not occur, and for this reason, no detrimental outcomes are observed. This hypothesis explains the differential effect of exogenous NO on the two types of grafts. Given such circumstances, it may be possible to develop some preventive strategies based on the use of NO donors for application in circumstances of non-steatotic grafts from DBDs used in LT; however, if steatosis is found to be present, then this same treatment would not be appropriate.

From our study, for the first time, we described signaling pathways underlying the I/R injury inherent to LT from DBDs that were specific depending on the presence or absence of steatosis in the liver graft. We showed that the fact that a liver is initially either steatotic or non-steatotic prior to graft collection may prove to be the determining factor for the endogenous signaling pathway. The fact that, in both types of liver grafts, we saw that proinflammatory and anti-inflammatory ILs were not well balanced as a result of BD induction could explain, at least partially, the detrimental effects induced by BD. Indeed, the results derived from the different pharmacological treatments indicate that the reduced ability to generate anti-inflammatory ILs (IL-6 in non-steatotic livers and IL-10 in steatotic ones) and the subsequent higher IL-1β levels in both types of liver grafts from DBDs were associated with the development of more severe liver injuries and inflammation, in both steatotic and non-steatotic LT from DBDs. Unfortunately, liver grafts from DBDs exhibit a decreased ability to endogenously generate such anti-inflammatory interleukins, as demonstrated by the results of the present study. This situation causes the grafts from DBDs to be unable to limit the high levels of IL-1β that were registered in both types of grafts, and consequently high levels of inflammation and damage occur.

The discovery of these mechanisms of action of IL-6 in non-steatotic livers and IL-10 in steatotic grafts allowed us to identify possible therapeutic targets whose effectiveness in reducing liver damage was evaluated in our research. Given such results and previous reports indicating that IL-1β is involved in the upregulation of reactive oxygen species (ROS) (15) and the production of inflammatory mediators (62, 126) observed in I/R liver injury, it could be useful to treat non-steatotic liver grafts with exogenous IL-6 or adopt a strategy based on supplementation with NO, and to treat steatotic ones with exogenous IL-10, with the purpose of limiting the inflammation and oxidative stress induced by IL-1β. Because of the results of the present research, the application of NO donors in steatotic liver grafts from DBDs would not be appropriate. Herein, we demonstrate a differential effect of NO supplementation depending on the type of liver graft from DBDs, since such a pharmacological strategy only protects non-steatotic grafts against damage through regulating the IL-6–IL-1β pathway, while in contrast, NO supplementation exacerbates oxidative stress, inflammation, and damage and does not affect the IL-10–IL-1β pathway in steatotic liver grafts.

In our view, it would be more appropriate to use pharmacological strategies with benefits in both types of livers (whether steatotic or non-steatotic), as there are no effective methods to distinguish between the presence and the absence of steatosis, or the degree of steatosis in the clinical context of DBDs (127). In such a case, given the preclinical results presented in the current study, the use of exogenous IL-6 or IL-10 and NO donors as therapeutic strategies may not be appropriate because of their specificity to only protect one type of graft and even NO donors can be prejudicial if steatosis is indeed present. Highlighting this type of disadvantage is extremely important for clinical surgical teams, because some studies have established that if a drug is useful in a model of hepatic I/R (whatever it may be), it could be used in all other settings involving liver injury from I/R (128–130). In this sense, the findings of the present study contribute to support the idea that, in the context of hepatic I/R, different therapeutic strategies have to be applied depending on each clinical context of I/R and dependently of the type of the liver (steatotic versus non-steatotic liver). Based on all of this, when performing LT, protection against the negative effects of BD may be provided by treatment adopting a strategy based on inhibiting the action of endogenous IL-1β, since such a strategy has been shown to be beneficial in liver grafts from DBDs both in the presence and in the absence of steatosis. The findings derived from the present investigation indicate that the administration of an IL-1β receptor antagonist would be an effective therapeutic strategy that potentially could be used in real clinical situations of LT from DBD. Since IL-1β inhibition has been shown to be equally effective in the presence or absence of steatosis, clinicians did not have to worry about knowing the degree of fatty infiltration that a liver graft that is going to undergo transplantation could have.

**Figure 12:**
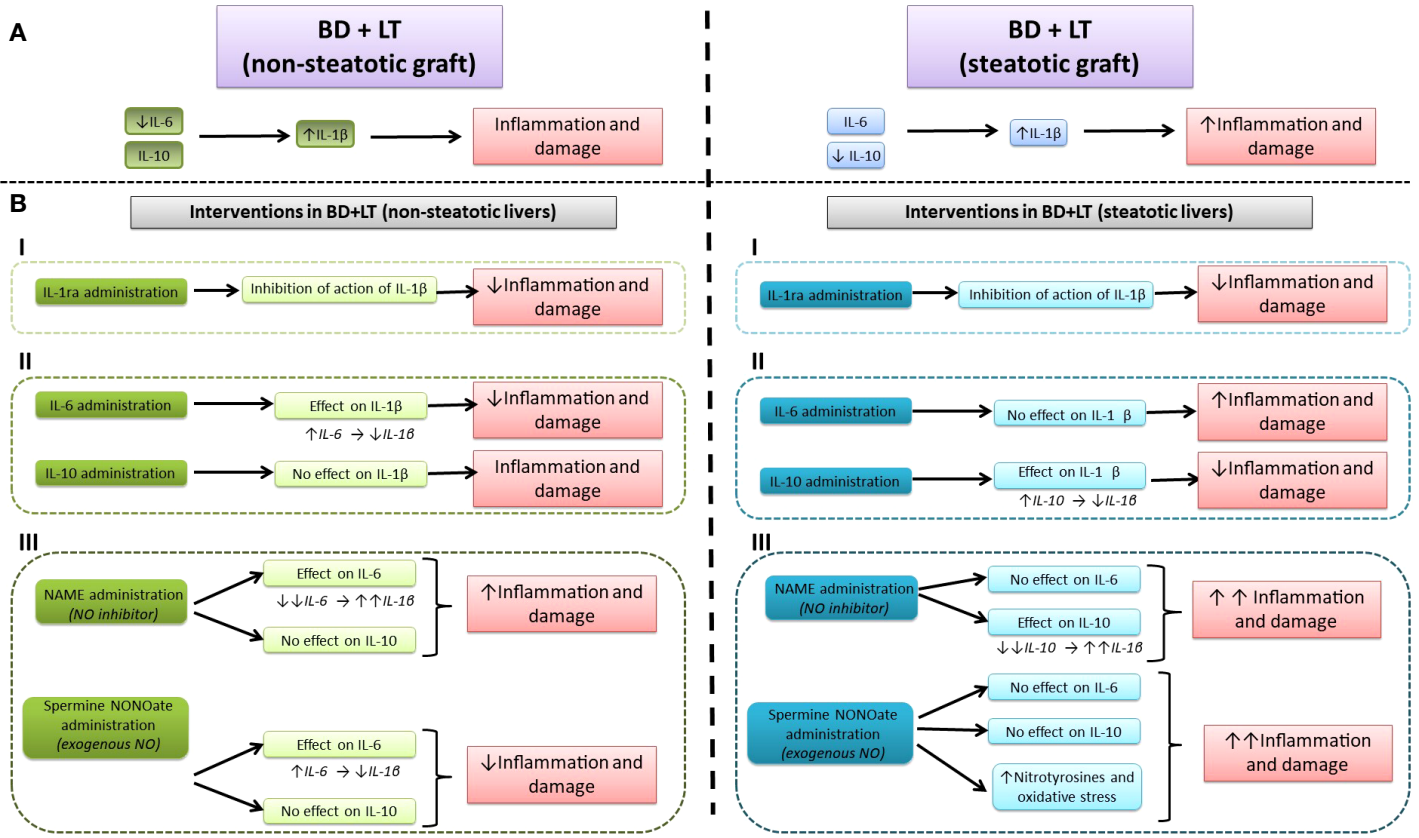
Signaling pathways highlighted by the current study. **(A)** Schematic representation of the role of interleukins in liver injury during transplantation with steatotic and non-steatotic grafts from DBDs. **(B)** Different pharmacological interventions: (I) effect of IL-1receptor antagonist, modulating the action of IL-1β; (II) effect of IL-6 and IL-10, modulating IL-1β levels; (III) effect of NO, modulating levels of IL-6, IL-10, and IL-1β.

In conclusion, results from our research showed that the fact that a liver is initially either steatotic or non-steatotic prior to graft collection may prove to be the determining factor for the signaling pathway in livers undergoing LT from DBDs. This is the NO/IL-6/IL-1b pathway in non-steatotic livers, and the NO/IL-10/IL-1b pathway in steatotic ones (summarized in **Figure 12**). From this, the usefulness of a therapy based on inhibiting the effects of IL-1β (using an IL-1 receptor antagonist) was demonstrated, which was effective in protecting against damage in both types of graft. Findings from the current study might be of considerable clinical relevance and could make a great contribution to the field of developing effective and efficient strategies that result in a reduction of the incidence of complications after LT in the case of grafts obtained from deceased donors, whether steatosis is present or not. In addition to the above, the present research established that the time frame between the declaration of BD and organ retrieval provides an important window for protective intervention, thus avoiding possible side effects in the recipient. From all of the above, it is clear that the results from the present investigation are of scientific and clinical relevance. Undoubtedly, further research beyond the scope of the present study will be necessary to determine whether the benefits of these strategies demonstrated in experimental models could reach clinical practice.

## Data availability statement

The raw data supporting the conclusions of this article will be made available by the authors, without undue reservation.

## Ethics statement

All procedures were approved by the Laboratory Animal Care and Use Committee of the University of Barcelona and by the Generalitat de Catalunya (DAAM 9353). European Union regulations (Directive 86/609 EEC) for animal experiments were respected.

## Author contributions

CP designed the study; CP, AC-R, and MM-C performed the groups of experiments, and biochemical and histological analyses; CP, AC-R, and MM-C contributed to data interpretation; AC-R, CM-S, and AS-G gathered the related literature and prepared the figures; AC-R drafted the manuscript; AT reviewed the manuscript and aided in its writing; CP provided a critical appraisal of the manuscript, expertise, and review. All authors contributed to the article and approved the submitted version.
